# Structural Maturation of HIV-1 Reverse Transcriptase—A Metamorphic Solution to Genomic Instability

**DOI:** 10.3390/v8100260

**Published:** 2016-09-27

**Authors:** Robert E. London

**Affiliations:** Genome Integrity and Structural Biology Laboratory, National Institute of Environmental Health Sciences, NIH, Research Triangle Park, NC 27709, USA; london@niehs.nih.gov; Tel.: +1-919-541-4879

**Keywords:** HIV-1 reverse transcriptase, reverse transcriptase maturation pathway, metamorphic protein, RNase H domain, subunit-specific RNase H domain unfolding, structural heterodimer

## Abstract

Human immunodeficiency virus 1 (HIV-1) reverse transcriptase (RT)—a critical enzyme of the viral life cycle—undergoes a complex maturation process, required so that a pair of p66 precursor proteins can develop conformationally along different pathways, one evolving to form active polymerase and ribonuclease H (RH) domains, while the second forms a non-functional polymerase and a proteolyzed RH domain. These parallel maturation pathways rely on the structural ambiguity of a metamorphic polymerase domain, for which the sequence–structure relationship is not unique. Recent nuclear magnetic resonance (NMR) studies utilizing selective labeling techniques, and structural characterization of the p66 monomer precursor have provided important insights into the details of this maturation pathway, revealing many aspects of the three major steps involved: (1) domain rearrangement; (2) dimerization; and (3) subunit-selective RH domain proteolysis. This review summarizes the major structural changes that occur during the maturation process. We also highlight how mutations, often viewed within the context of the mature RT heterodimer, can exert a major influence on maturation and dimerization. It is further suggested that several steps in the RT maturation pathway may provide attractive targets for drug development.

## 1. Introduction

The genome of RNA viruses is subject to severe size limitations that result from the chemical instability of the RNA polymer [1] and from biological instability attributed to reliance on low-fidelity polymerases [2]. Human immunodeficiency virus 1 (HIV-1) reverse transcriptase (RT) exhibits such poor fidelity that the virus is believed to function on the threshold of an error catastrophe [3,4]. As a consequence of these constraints, the HIV-1 genome is only 9.2 kb [5], about average for RNA viruses [2,6]. Compression of the HIV genome is achieved by several strategies, including the use of polycistronic coding sequences dependent on a programmed ribosomal frameshift [7,8,9]. Remarkable as this strategy is, the most significant size limitation of the viral genome probably results from limiting the size of the required viral proteome. RNA viruses utilize two major structural approaches to limit the size of the proteome: (1) multifunctional proteins, and (2) symmetric, multisubunit proteins. Ignoring size variations, a protein that fulfills *n* functions produces an *n*-fold reduction in the coding requirement. The accessory proteins negative regulatory factor (Nef) [10,11] and viral protein unique (Vpu) [12], as well as HIV RT, which possesses RNA-dependent DNA polymerase, ribonuclease H (RNase H), and DNA-dependent DNA polymerase activities [13], all provide examples of multifunctionality. Active HIV protease (PR) is dimeric [14], and HIV integrase (IN) functions as a tetramer [15], similarly achieving *n*-fold reductions in coding, where in this case, *n* represents the number of symmetric units in the functional molecule. HIV-1 RT utilizes a third strategy—a metamorphic polymerase domain for which the sequence–structure relationship is ambiguous; the sequence does not correspond to a unique three-dimensional fold. The ability of the domain to adopt alternate structures, each of which fulfills a distinct function, represents another approach to expanding the functionality of the proteome while limiting the size of the corresponding genome.

The metamorphic nature of the RT polymerase domain also adds a layer of complexity to the maturation process, required so that a pair of p66 precursor proteins can develop along different pathways, one evolving to form active polymerase and ribonuclease H (RH) domains, while the second develops along a pathway leading to a non-functional polymerase and to proteolysis of its associated RH domain. Recent studies have revealed that the alternative fold of the polymerase domain also plays a role in unfolding the second, supernumerary RH domain that is present in the initially formed homodimer [16]. Delineating the steps involved in the conformational maturation pathway of RT has proceeded much more slowly than the determination of its three-dimensional structure. However, a combination of nuclear magnetic resonance (NMR), crystallographic, and molecular modeling studies have provided information revealing the general features of the maturation process. This pathway involves three basic steps: (1) domain rearrangements; (2) dimerization; and (3) subunit-selective RH domain proteolysis. To a large extent, the order of these operations is not commutative; there is a functional dependence of each step on the preceding step.

Although RT-directed therapeutic agents currently in use target the mature RT heterodimer [17,18,19], reliance on a complex maturation process introduces the possibility of targeting the enzyme at various points along its maturation pathway. In principle, such maturation-targeted agents might be expected to have a reduced tendency to interact non-specifically with other cellular polymerases, as occurs, for example, in nucleoside inhibitors [20]. Additionally, the metamorphic nature of the RT molecule has important implications for its ability to tolerate useful mutations and for understanding how mutations can influence RT behavior. In contrast with fully symmetric proteins, the RT heterodimer contains regions of differential mutational sensitivity, providing somewhat greater evolutionary flexibility. In this review, we outline recent progress toward understanding the RT maturation pathway.

## 2. Structural Background

### 2.1. Nomenclature and Constructs Studied

Reverse transcriptase has two functional domains, polymerase and RNase H, with the polymerase made up of fingers (F), palm (P), thumb (T), and connection (C) subdomains, although some authors define the polymerase domain to include only the F, P, and T subdomains (Table 1). In order to simplify the presentation, the rigorous distinction of domain vs. subdomain has been ignored in this review. Domain boundaries are defined as: F (1–84; 119–154); P (85–118; 155–241); T (242–313); connection (314–426); RH (427–560). These are generally similar to the domain boundaries defined by Ding et al. [21] and Kohlstaedt et al. [22], with the main differences being the earlier termination of the T domain (which simplifies the description of the metamorphic transformation), and the connection:RH boundary. This RH domain boundary corresponds to the minimum RH construct required to form a stable protein that can be separately isolated and studied [23]. Since the sequences of the F and P domains are discontinuous, the domains cannot dissociate, and the combined domain is abbreviated as F/P. Domain complexes are abbreviated using a colon, such that, for example, “F/P:connection” represents the complex formed from the fingers/palm and the connection domains.

The complexity and sequence degeneracy of the RT homodimer requires particular attention to the nomenclature required to distinguish between the sequence-identical subunits. As in previous publications, we have used the structure-dependent labeling introduced previously [25], in which we designate p66*M* as the *monomeric* structure; p66*E* contains the more *extended*, actively-folded polymerase domain observed in the p66 subunit of the RT heterodimer; p66*C* corresponds to the p66 subunit of the homodimer that contains the *compact* and inactively folded polymerase domain (p51*C*) linked to a separate RH domain. The p66*E* and p66*C* structures each exhibit additional conformational flexibility. The fingers and thumb domains of p66*E* preferentially adopt a closed conformation in the absence of ligands [26], and an open conformation when complexed with double-stranded nucleic acid substrates or with non-nucleoside RT inhibitors (NNRTIs). The p66*C* structure is subject to even greater conformational variability, developing from a p66*M*-like conformation in the initially formed homodimer (described in greater detail below) to a conformation in which the polymerase domain conformation becomes equivalent to the p51 subunit of the heterodimer and the associated RH domain is unfolded. The p66*C* conformational transitions that occur within the context of the p66*C* structure are initiated by dimer formation and transpire over a slower time frame.

Subsequent to dimer formation, the structural differences between the subunits allows the introduction of the p66/p66’ nomenclature, in which the non-primed subunit contains the active polymerase and RH domains, and the primed subunit becomes committed to developing into the inactively-folded polymerase’ plus an unfolded RH’ domain. Crystal structures representative of these conformations and related structures discussed in this review are summarized in Table 2.

### 2.2. Structural Features of the RT Heterodimer

**Figure 1:**
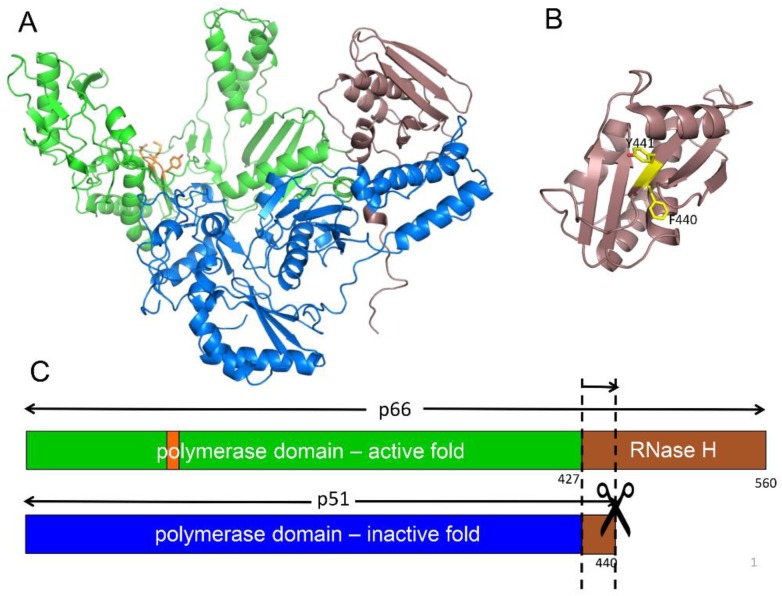
Structure of the reverse transcriptase (RT) heterodimer. (**A**) Ribbon diagram of p66/p51 showing the active polymerase (green) and ribonuclease H (RNase H, brown) domains in p66, and the inactive polymerase (blue) and RNase H domain fragment, residues 427–440 (brown), in the p51 subunit. The YMDD motif at the active site in p66 is shown in orange; (**B**) Structure of the isolated RNase H domain identifying the buried Phe440–Tyr441 cleavage site (yellow); (**C**) Domain structure of the two RT subunits color coded as in A, and illustrating the internal cleavage site that produces the p51 subunit.

The active form of HIV RT is a p66/p51 heterodimer in which the p51 subunit has been derived from one p66 subunit by proteolytic processing of an occult cleavage site located within the RH domain (Figure 1). The active polymerase and RNase H sites are both located on the p66 subunit, while in the p51 subunit, the polymerase adopts an alternative, inactive fold and is thus devoid of enzymatic activity. Importantly, the HIV protease cleavage site within p66 is not located at the domain boundary, but at an internal position within the RH domain (Figure 1B,C). Although it has occasionally been reported that the virion contains an active RH monomer [35], most studies indicate that internal cleavage of the domain at Phe440–Tyr441 produces an unstable fragment that is subject to rapid further degradation and hence does not yield an active RH [23,36,37]. The conclusion that RH(441–560) is unstable is further supported by more recent studies indicating that the deletion of N-terminal Tyr427 and Gln428 residues results in significant destabilization of the isolated RH domain [16], and that even single mutations in the 427–440 segment preceding Tyr441 result in partial or complete RH domain unfolding [38,39].

The buried location of the cleavage site poses an interesting problem in conformational maturation: cleavage must occur either prior to folding of the RH domain or subsequent to RH domain unfolding. The main difficulty with the first alternative is the requirement of a regulatory mechanism that will limit cleavage so that only 50% of the p66 chains are proteolyzed. Indirect support for an unfolding step in the maturation pathway has come from studies demonstrating that p66 containing mutations that reduce dimer formation also inhibit proteolytic formation of p51 [40,41]. This result supports the conclusion that formation of the p66/p66’ homodimer results at some point in the formation of a structural heterodimer that presumably exposes the Phe440–Tyr441 cleavage site in one of the subunits. Recent NMR studies have characterized the p66/p66’ homodimer as a structural heterodimer, and demonstrated that this asymmetry results in subunit-selective unfolding of the RH’ domain on a single subunit [16,24].

### 2.3. The Alternate Folds of the Polymerase Domain

**Figure 2:**
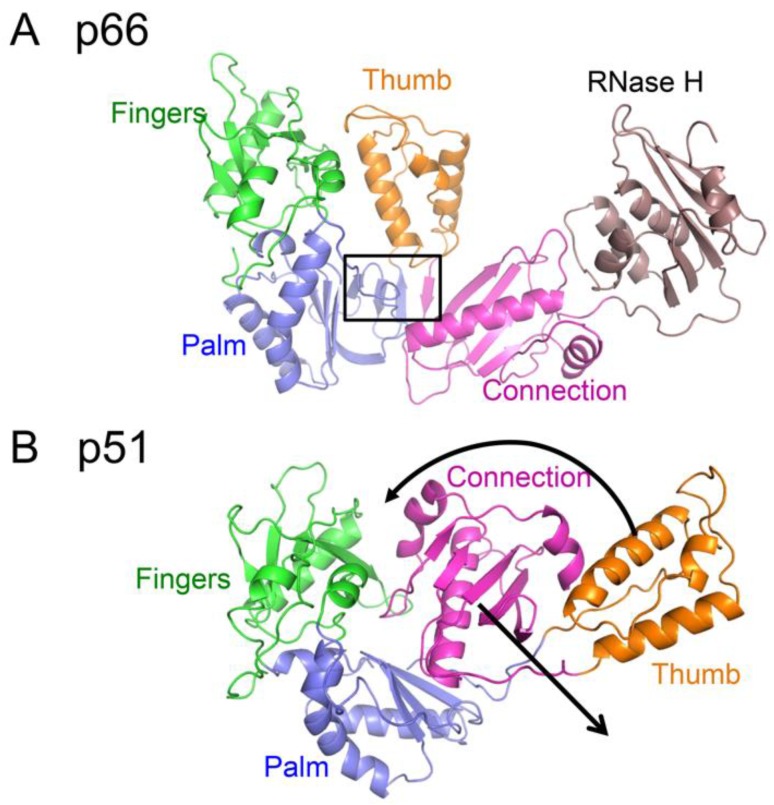
Alternative structures of the metamorphic polymerase domain. (**A**) The *extended* structure observed in the p66*E* subunit of RT; (**B**) the *compact* structure observed in the p51 subunit of the RT heterodimer. Color coding is: fingers (green); palm (blue); thumb (orange); connection (magenta). The position of the RH domain in the p66 subunit is also shown (brown), although this domain does not participate in the metamorphic rearrangement. The extended conformation includes a short, four-stranded β-sheet that forms part of the palm:connection interface in the *extended* conformation (boxed) that is not present in the *compact* conformation. Black arrows indicate the major domain rearrangements that need to occur for the metamorphic transition.

The polymerase domain of RT is an example of a metamorphic protein (i.e., a protein characterized by an ambiguous relationship between sequence and structure), so that the sequence does not strongly favor a single, unique fold [42,43]. Two alternate folds of the polymerase domain that are present in the two subunits of the RT heterodimer are shown in Figure 2. Most proteins exhibit substantial conformational flexibility that is related to conformationally-dependent interactions, substrate binding and release, and to optimization of the catalytic site in order to facilitate catalysis. Large conformational changes generally accompany the interaction of polymerases with their substrates [44,45,46]. Crystallographic studies of RT–substrate complexes reveal that in RT, the thumb domain must undergo a large repositioning to accommodate the double stranded DNA (dsDNA) or RNA–DNA substrate [27], and the fingers domain closes down over the ternary DNA–dNTP complex [47]. However, these conformational changes generally require only bond rotations and are relatively modest in comparison with the domain rearrangements and changes in secondary structure that differentiate the structures of the polymerase domains in the p66 and p51 subunits (Figure 2). Interconversion of these structures requires the dissolution and formation of several interfaces as well as changes in secondary structure, particularly involving residues near the C-termini of the palm and connection domains [21]. Formation of the palm:connection interface in the active polymerase requires the formation of a four-stranded β-sheet not present in the monomer (Figure 2).

Although for most metamorphic proteins that have been studied, transition from one form to the other is triggered by altering the conditions under which they are characterized, both metamorphic forms of p66 must exist under the same set of physiological conditions in order to form the structural heterodimer. The less stable *extended* conformation is ultimately stabilized by the formation of a large dimer interface of nearly 5000 Å^2^ [21,48].

### 2.4. Structural Characterization of the p66 Monomer

The p66 monomer is the initial substrate for the maturation process, so its structural characterization is extremely useful for understanding the initial steps in the maturation process. Further, for reasons outlined above, it is important to determine whether the RH domain in p66*M* adopts an unfolded, partially folded, or fully folded structure. Since p66 has a strong tendency to homodimerize [49,50,51], p66*M* characterization requires the imposition of conditions or the introduction of mutations that will preclude or strongly limit dimer formation. Although multiple factors, including experimental conditions, mutations, and various ligands have been identified that reduce dimer formation, the relatively high concentrations required for NMR or crystallographic characterization require the use of a strategy that functionally blocks dimer formation. An approach for achieving this result was suggested by an early analysis of the heterodimer structure by Wang et al. [48], indicating that in isolation, the p66 monomer would adopt a compact structure similar to that observed in the inactive p51 subunit of RT. This conclusion was based primarily on the large reduction of solvent-accessible hydrophobic surface in the p51 polymerase domain, relative to the p66 polymerase domain. A structural comparison of the *extended* and *compact* polymerase domains (Figure 2) reveals the presence of a disordered loop corresponding to residues 219–230 in the inactive polymerase domain. Since the residues are disordered, deletion of this loop would not be expected to significantly perturb the p51 subunit of RT or—based on the above hypothesis—the structure of the polymerase monomer. In contrast, this segment fulfills structurally and functionally important roles in the active polymerase domain, forming part of the primer grip motif [52]. Loop deletion thus targets domain rearrangement more directly than many other mutations reported to interfere with dimer formation that are frequently located at or near the subunit interface.

**Figure 3:**
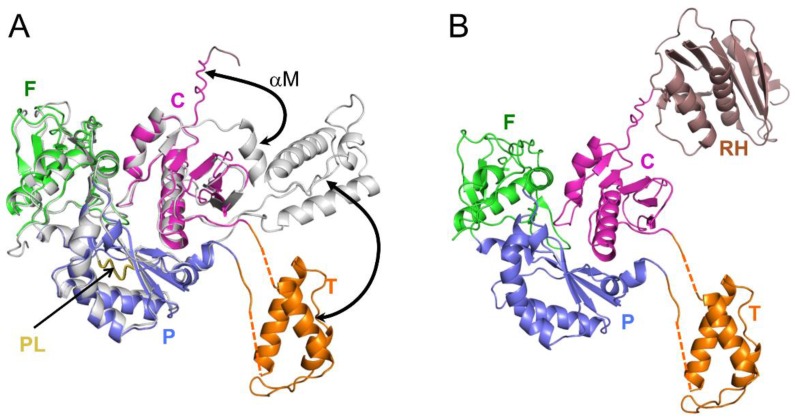
The p51 and p66 monomer structures. (**A**) Ribbon diagram of the p51∆PL monomer overlaid with the p51 subunit of RT (PDB ID: 1S9E). Structure 1S9E (gray) corresponds to an inhibitor complex, and includes the palm (P) loop segment (residues 219–230), which forms a loop at the rear of the structure (gold). The two major differences relative to the p51 subunit of RT are the disordered position of the thumb (T) and the C-terminal residues 420–430, which form helix αM’ in the p51 subunit; (**B**) Ribbon visualization of the p66 monomer based on the crystal structure shown in panel A and nuclear magnetic resonance (NMR) data indicating resonances of the RH domain in close agreement with those of the isolated domain. The unfolded C-terminus of the connection (C) domain in A links the polymerase and RH domains. Domain color coding as in Figure 2.

A construct of the polymerase domain lacking palm loop residues 219–230 (subsequently referred to as p51∆PL) was expressed and crystallized as a monomer [16]. Overlaid ribbon diagrams for the p51∆PL monomer (color coded by domain) and the p51 subunit of RT (gray) are shown in Figure 3A. The crystal structure of *monomeric* p51∆PL reveals a domain orientation pattern that is similar to that of the p51 subunit of RT, while exhibiting two significant differences: (i) The C-terminal residues of the connection domain corresponding to residues ~421–430, which form helix αM’ in the p51 subunit of RT, are unfolded; and (ii) the three-helix bundle that forms the core of the thumb remains intact, but the two segments that link the thumb to the palm and connection domains are disordered, so the orientation of the thumb is not fixed. Observation of the thumb and disordered helix M residues in the crystal structure was a fortuitous result of stabilizing lattice contacts. Given the interaction of helix M with the thumb domain in the p51 subunit of RT (Figure 3A), it is very likely that positioning of the thumb and formation of helix αM are cooperative processes that are facilitated by dimer formation and formation of the intersubunit RH:thumb’ interface [27]. Zheng et al. also reported NMR studies supporting the conclusion that the observed *monomeric* structure did not arise as an artifactual consequence of the palm loop deletion [16].

These structural results for the p51 monomer were extended to an analysis of the structure of p66*M* based on NMR spectroscopic comparisons of Ile-labeled constructs containing the L289K mutation in order to reduce dimer formation [16]. These comparisons show that the NMR spectrum of a sample containing both the labeled p51 and isolated RH domains closely approximates the spectrum of the p66 monomer [16]. This comparison indicates that in solution, p66*M* adopts a structure that includes a p51*M* domain linked to a folded RH domain in a way that produces very few resonance changes relative to the spectra of the isolated domains. The crystal structure of p51∆PL provides an immediate solution for satisfying this constraint, since residues at the C-terminus of the p51∆PL are disordered, and thus can form a linker with the folded RH domain (Figure 3B). In addition, this analysis indicates that the RH domain remains in a folded state with its Phe440–Tyr441 cleavage site inaccessible to the protease. Thus, similar to the isolated RH domain [36,53], p66*M* is expected to be resistant to proteolysis of the cleavage site.

## 3. The Metamorphic Transition of the Polymerase Domain

### 3.1. Monomer Domain Dissociation

As outlined above, the p66 monomer adopts a conformation that approximates that of the p51 subunit of RT, but differs significantly from that of the active polymerase domain in the p66 subunit. Three key observations support domain isomerization as the first step of conformational maturation. (1) The loose organization of the monomer (Figure 3) suggests that it is prone to unimolecular domain reorganization. Thus, the thumb and RH domains lack interface contacts, and only a single interface corresponding to the fingers/palm:connection is present. Therefore, the domain rearrangements required to form the *extended* polymerase conformation might be viewed as already in progress; (2) mutations that block domain rearrangements indirectly block dimer formation. The palm loop deletion described above was developed to block isomerization of the p66 *monomeric* to its *extended* conformation (Figure 2A). The deleted residues are not located near the dimer interface, but chromatographic analysis demonstrates that p66∆PL does not dimerize [24]. Similarly, other p66 subunit mutations, including L234A and L234E, that (as outlined in a later section) strongly inhibit the *M→E* conformational transition also strongly reduce dimer formation, while the same mutations produce no significant effect if introduced into the p51 subunit [41,54]. These observations support the conclusion that a subunit rearrangement is required for dimer formation; dimerization is limited by the requirement for a prior isomerization step; (3) As discussed by Kohlstaedt et al. [22], dimer formation is limited by the availability of a required dimerization surface—the hydrophobic surface of the connection domain. The inaccessibility of this surface in the RT heterodimer prevents it from forming higher aggregates. The dimerization surface of the connection domain in the monomer becomes available as a consequence of initial unimolecular domain rearrangements that are described below [24]; (4) Although early kinetic studies indicated rapid dimer formation followed by slower conformational adjustments (e.g., [55]), more recent studies indicate that initial dimer formation is considerably slower, consistent with a requirement for prior domain rearrangements [56].

**Figure 4:**
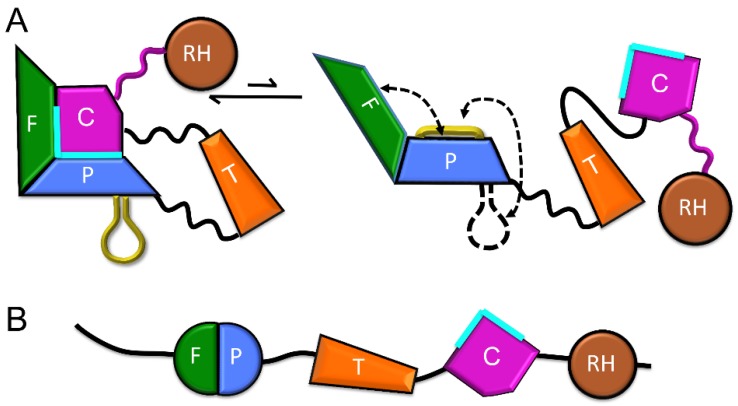
Schematic representation of fingers/palm:connection dissociation in p66*M*. (**A**) The structure of p66*M* approximates a domains-on-a-string model in which a few of the domains—corresponding to the fingers/palm:connection cluster—remain associated. Subsequent to dissociation, the non-dissociable fingers/palm domains adopt an altered, more extended conformation, in which the initially disordered palm loop (gold) is recruited to the inner surface of the palm domain. These conformational changes reduce the tendency toward reassociation, introducing kinetic barriers that extend the lifetime of the fully dissociated species. Dissociation exposes the hydrophobic surface of the connection (C) domain (cyan); (**B**) Beads-on-a-string model of the fully domain-dissociated monomer, in which the hydrophobic surface of the C domain (cyan) provides the initial interaction surface for dimer formation with a second, non-dissociated p66*M* molecule. Domain color coding as in Figure 2.

**Figure 5:**
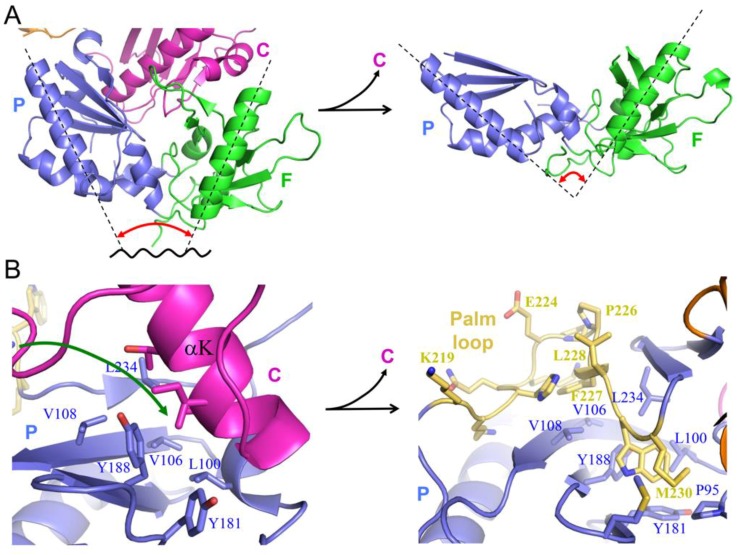
Structural changes of fingers/palm (F/P) that follow fingers/palm:connection dissociation. (**A**) Dissociation of the monomer fingers/palm:connection interface (PDB ID: 4KSE) leads to an expansion in the fingers/palm angle for the isolated fingers/palm domain (PDB ID: 1HAR); (**B**) Dissociation of the fingers/palm:connection interface in the monomer gives the palm loop access to the inner face of the palm domain, where it interacts with the same surface that forms the palm:connection interface (PDB ID: 1DLO). Domain color coding as in Figure 2.

The p66*M* structure can be described as beads on a string, in which a few of the beads corresponding to the fingers/palm:connection complex have become stuck together. Nevertheless, the fingers/palm:connection interface, while highly stable, remains subject to occasional dissociation (Figure 4). Recent analyses based on NMR studies of Ile-labeled RT, crystal structure data, and molecular modeling have revealed that once the fingers/palm:connection domains have dissociated, several conformational adjustments occur that reduce the likelihood of immediate reassociation [24]. These adjustments include expansion of the fingers/palm domain, as defined, for example, by the angle between helices A (fingers domain) and F (palm domain) (Figure 5A), which increases from ~45° to ~90°, and the movement of the initially disordered palm loop to the inner surface of the palm domain (Figure 5B). The palm loop contacts many of the same hydrophobic residues that interact with connection domain helix αK in the *monomeric* structure (Figure 5B). One driver for this conformational change of the fingers/palm may be elimination of the bend in helix αE near residue Phe160 that is present in the *monomeric* structure. Re-formation of the more stable *monomeric* structure is then limited by the rate of dissociation of the palm loop from the inner surface of the palm domain. We emphasize that the fingers/palm:connection complex illustrated in the upper left of Figure 4 represents the most thermodynamically stable form. The conformational changes illustrated in Figure 4 and Figure 5 will presumably retard the rate of domain reassociation. By extending the lifetime of the fully domain-dissociated monomer, these adjustments allow the connection domain time to make intermolecular contacts so that a dimer can be formed.

Interestingly, this same hydrophobic surface of the palm domain that is able to interact with hydrophobic surfaces on either the connection domain or on the palm loop also forms part of the NNRTI binding site [57,58]. Thus, the same adaptability supporting the conformational changes required for structural maturation of the RT molecule also supports binding of a broad range of hydrophobic NNRTI ligands.

### 3.2. Formation of New Interfaces in the Extended Conformation

**Figure 6:**
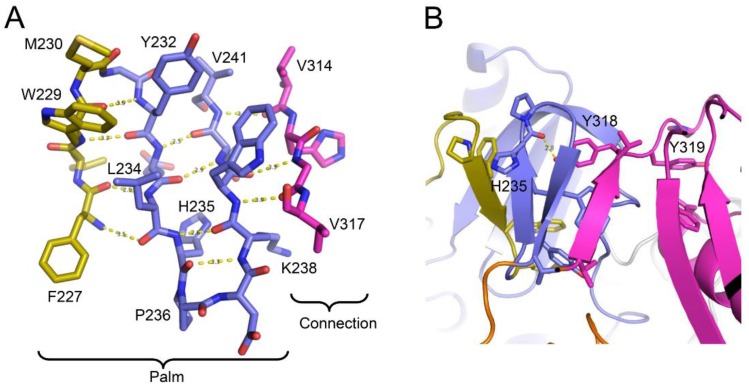
Formation of the palm:connection interface in p66*E*. (**A**) β-sheet structure formed from palm loop residues (gold) and following palm domain residues 231–241 (blue), and residues 313–317 in the connection domain (magenta), that form the new palm:connection interface; (**B**) Formation of the β-sheet also creates a binding pocket for connection domain residue Tyr318, which also forms an H-bond with His235.

The *extended* polymerase conformation that is observed in the RT heterodimer contains several interfaces that are not present in the *monomeric* structure, and thus need to be formed as part of the maturation process. In general, formation of the *extended* conformation requires large structural changes in the palm and connection domains, and relatively smaller adjustments of the fingers, thumb, and RH domains. Recruitment of the palm loop to the inner surface of the palm domain described above (Figure 5B) stabilizes an *extended* segment (residues Phe227–Met230) that facilitates accretion of additional β-strands, ultimately forming a four-stranded β-sheet that includes the entire C-terminal segment of the palm domain, Phe227–Val241, and finally connection domain residues Val314–Val317 (Figure 6A). These changes further illustrate the critical adaptability of the palm loop and subsequent segment to adopt alternate secondary structures that facilitate the formation of alternate interface contacts; strands 1 and 4 of the β-sheet shown in Figure 6A are formed from segments that are disordered in p66*M* (palm loop residues 219–230 and connection residues 313–315) (PDB ID: 4KSE). The loop → β-sheet transition is initiated by domain dissociation and is driven by the intrinsic structural preferences of the residues. Formation of the β-sheet structure is facilitated by residues at the turn positions that favor β-turn structures. Both of the β-turn sequences: Trp229-Met-Gly-Tyr and, to a greater degree, His235-Pro-Asp-Lys, score above the cutoff value used by Chou and Fasman to define probable β-turns [59]. The β-sheet structure is reinforced by an extensive network of hydrophobic interactions that include residues Phe227, Trp229, Met230, Tyr232, Leu234, Trp239, Val241, and Val314 (Figure 6A). These side chain interactions play an important role in stabilizing the β-sheet structure which is necessary to drive the *M→E* transition, and their role in RT maturation is further supported by mutational results discussed later in this review.

Formation of this short β-sheet also creates a binding pocket for connection residue Tyr318 that contributes to a stable palm:connection interface (Figure 6B). The Tyr318 hydroxyl forms an H-bond with the His235 backbone and interacts hydrophobically with Leu100, Lys102, Leu234, and Trp239. The side chain of Tyr319 extends in the opposite direction, so that the consecutive Tyr residues penetrate deep into the palm and connection domains, stabilizing the palm:connection interface (Figure 6B).

**Figure 7:**
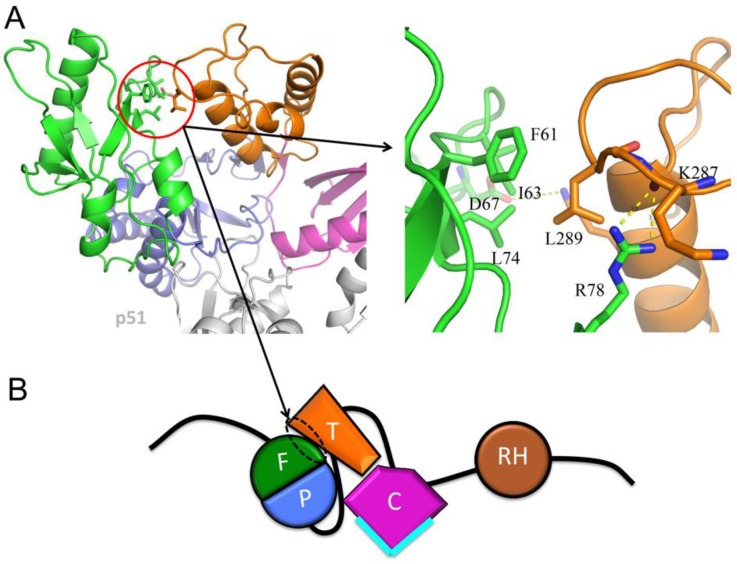
The fingers:thumb interface in apo RT. (**A**) Ribbon diagram showing Leu289 and other residues at the fingers:thumb interface in the p66 subunit of apo RT (PDB ID: 1DLO) (**left**), and expanded view of interface interactions (**right**); (**B**) Schematic illustration of the domain organization facilitated by the formation of the fingers:thumb interface during the maturation process. Domains are color coded as in Figure 2 and Figure 4.

A small fingers:thumb interface is present in the p66 subunit of apo RT (Figure 7), PDB ID: 1DLO, [27]. This interaction appears to position the thumb domain so that it does not interfere with—and may optimize—the relative positions of the palm and connection domains in order to facilitate formation the fourth strand of the β-sheet. Given the position of Leu289 at this interface, this hypothesis provides an attractive explanation for the effect of the L289K mutation on dimer stability [25,60], and for the unusual closed fingers–thumb structure of the apo enzyme, in which the substrate binding site is occluded.

**Figure 8:**
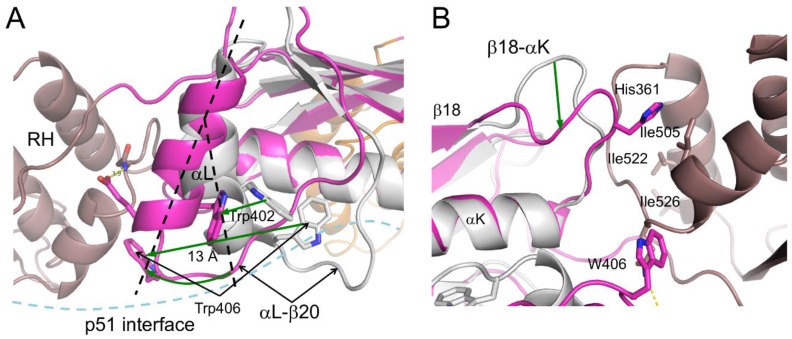
Formation of the connection:RH interface. (**A**) Superposition of the connection:RH interface region of the p66 subunit of the RT heterodimer (color coded as in Figure 2) with the monomer connection domain (gray). Formation of the connection:RH interface requires straightening of helix αL, which also requires movement of the αL–β21 loop. These adjustments are required to form both the intra-subunit connection:RH interface and the inter-subunit connection:connection’ interface. Trp406 moves approximately 13 Å to make contact with the RH domain (the α-carbon displacement is 11 Å); (**B**) Rotated view illustrating movement of the β18–αK loop and conformational changes at the connection:RH interface that result in several of the observed RH domain Ile shifts previously reported [24]. Black hyphenated lines indicate the altered helix structure, and green arrows indicate conformational changes.

More extensive conformational changes in the connection domain occur at the connection:RH interface. Formation of this interface requires straightening of helix αL and changes in the position of the Trp-rich loop that follows αL, bringing Trp406 into contact with the RH domain (Figure 8A). The adjustments of this loop are also required to form the subunit interface with p51 (or p66’) (not shown), suggesting a cooperative process. A favored scenario could involve initial connection domain adjustments at the subunit interface, cooperatively facilitating the formation of the p66 connection:RH interface, which in turn would facilitate subsequent formation of the inter-subunit RH:thumb’ interface. There is also a significant conformational change of the β18–αK loop, which positions connection residue His361 near RH residue Ile505 (Figure 8B) [24]. At the present time, it is unclear to what extent these adjustments of the connection domain are unimolecular conformational changes that follow domain dissociation and to what extent they result from induced fits at the domain and subunit interfaces. To the extent that the domain boundaries are dynamic, these adjustments may also occur by a conformational selection process. Several observations suggest that they are more likely to involve cooperative formation of several different connection domain interfaces ([24] and unpublished simulations).

## 4. Dimerization-Induced Structural Changes

### 4.1. Dimer Formation

The monomer domain dissociation described above (Figure 4 and Figure 5) releases the connection domain from its interface with the fingers/palm domains, allowing it to form a dimer with a second monomer. This may be considered as a conformational selection process in which the predominant monomer selects the rare species with the free connection domain to form an initial dimer. Hydrophobic interactions tend to be relatively strong but non-specific—ideal for formation of an initial interface that can then adjust conformationally to include additional, more specific interactions. As noted above, adjustments of helix αL and the following αL–β21 loop facilitate the formation of both the intra-subunit connection:RH and the intersubunit connection:connection’ interfaces, and may form cooperatively (Figure 8A). Dimerization thus more closely corresponds to a hybrid process that may be most accurately described as an initial selection by the monomer for molecules with an available connection domain, followed by additional induced conformational adjustments.

**Figure 9:**
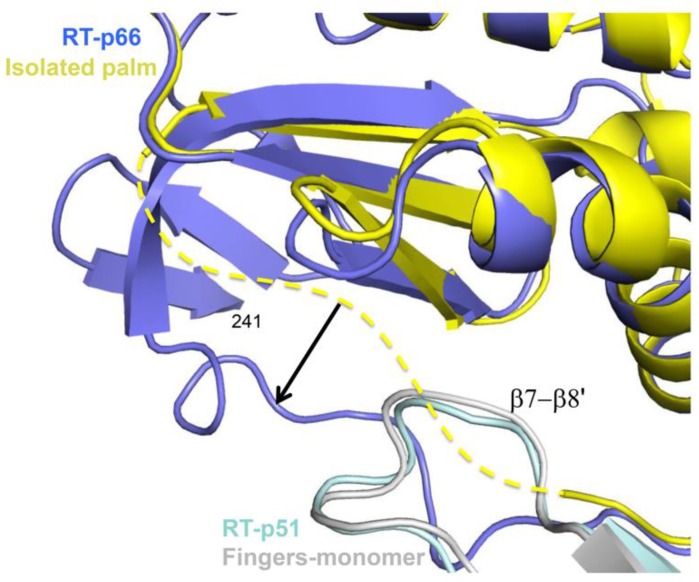
Formation of the palm:fingers’ interface. Superposition of the inter-subunit palm:fingers’ interface in the unliganded heterodimer (PDB ID: 1DLO) (purple:pale green) with the corresponding precursor structures: the isolated palm domain (yellow, PDB ID: 1HAR) and the monomer fingers domain (gray, PDB ID: 4KSE). The palm segment from 91–103 that is disordered in the crystal structure of the isolated fingers/palm construct becomes ordered in the heterodimer structure, presumably as a result of an induced fit process. Structural comparison of the β7–β8 loop in the fingers domain of the monomer (PDB ID: 4KSE) with the heterodimer p51 subunit (PDB ID: 1DLO) indicates that only minimal adjustments are required to form the dimer interface.

In addition to the structural changes in the palm domain required to form a new intra-subunit interface with the connection domain described above, the surface of the palm domain that contacts the fingers’ domain in the p66’ subunit of the p66/p66’ homodimer must also undergo additional conformational adjustments. Development of the mature palm conformation corresponds to progression through structures characterizing the monomer (PDB ID: 4KSE, [16]), the isolated fingers/palm construct (PDB ID: 1HAR, [29]), and ultimately the mature heterodimer (PDB ID: 1DLO, [27]). Structural comparisons indicate that the palm domain segment from residue 90–103 located at the subunit interface becomes more disordered in going from the monomer to the isolated fingers/palm construct. Subsequent formation of the subunit interface of the palm domain on p66 involves an induced fit interaction with the β7’-β8’ loop in the fingers’ of p66’, which has been shown to contribute significantly to dimer formation (Figure 9) [61,62].

Importantly, all of the NMR spectra that have been obtained for the Ile-labeled p66/p66’ homodimer reveal many subunit-specific chemical shift differences, consistent with the conclusion that the p66/p66’ homodimer exists as a structural heterodimer, and consistent with the above conformational selection process in which two conformationally distinct p66 monomers form an initial homodimer. Fortuitously, despite their close structural similarity, the RH and RH’ domains initially present in the p66/p66’ homodimer yield many separate resonances, allowing their separate conformational evolution to be individually tracked [16]. In contrast with these conclusions and other studies supporting an asymmetric p66/p66’ homodimer structure [48,63], recent NMR studies of U-[^2^H,^15^N]p66 have been interpreted to indicate the formation of a stable, symmetric p66/p66 homodimer containing two structurally and magnetically equivalent, stable RH domains [64]. However, recent NMR studies of [^2^H,^15^N]-labeled p66 in our lab obtained under conditions similar to those of Sharaf et al. [64] are dominated by resonances attributable to the smaller and more flexible p66 monomer, rather than to the homodimer (unpublished data).

### 4.2. Maturation of the p66’ Subunit Involves a Molecular Tug-of-War

**Figure 10:**
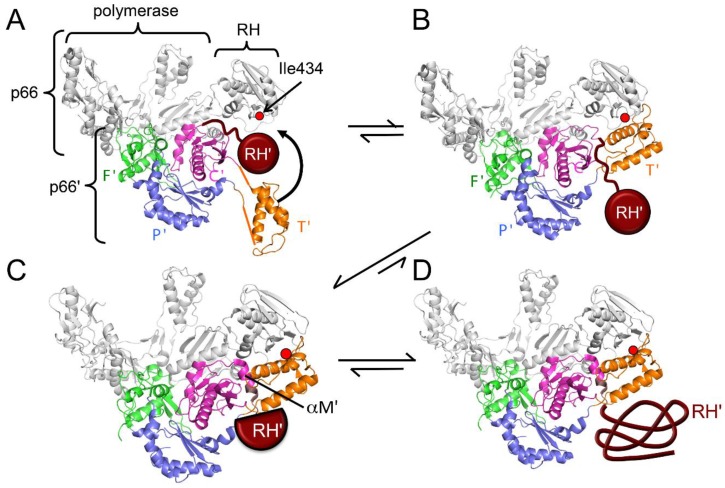
Maturation processes that follow dimer formation. (**A**) Schematic ribbon diagram of an early monomer in which the p51 subunit of the heterodimer has been replaced by p66*M* (Figure 4B) and the RH’ domain is represented by a brown circle; (**B**) Schematic of the RT heterodimer subsequent to formation of the RH:thumb’ (T’) interface and repositioning of the connection’ (C’) linker and RH’ domain; (**C**) Schematic showing a more mature form of the homodimer in which helix αM’ is fully formed and RH’ has become destabilized as a result of the loss of several N-terminal residues; (**D**) Schematic of the final structure of the p66/p66’ homodimer after RH’ unfolding. In the above structures, the p66 subunit is shown in gray and the domains of the p66’ subunit are color coded as in Figure 2. Although no RT ligands are assumed, the p66 subunit conformation is based on a structure with an open fingers (F’)/thumb (T’) conformation for clarity. Transitions of the polymerase’ domain in the p66’ subunit correspond to the *monomeric* (*M*)*→compact* (*C*) structures shown in Figure 3A. The location of the p66 Ile434 residue—which provides a resonance sensitive to the formation of the RH:T’ interface—is indicated.

According to the conformational selection model presented above, the p66’ subunit in the initially formed p66/p66’ homodimer is expected to be very similar to p66*M* (Figure 3B). Consequently, no interface contacts are expected for either of the weakly tethered thumb’ and RH’ domains in the initial homodimer (Figure 10A). Zheng et al. [24] have demonstrated that the δ-methyl shift of Ile434—located in the RH domain at the RH:thumb’ interface—is sensitive to the structure of this interface, and thus can serve as a useful monitor for its formation. NMR studies performed during the first five-hour period after the introduction of dimer-favoring conditions revealed that the inter-subunit RH:thumb’ interface had formed early in this period, probably within the first 30 min, and perhaps on a considerably shorter time scale [24]. The NMR studies further indicated that the formation of this interface appears to be cooperative with the formation of the connection:RH interface, and perhaps other interface contacts within the p66 subunit. Therefore, the initially formed p66/p66’ homodimer undergoes early conformational adjustments that include formation of the RH:thumb’ interface and are largely completed over a time span much shorter than the accumulation period of 5.5 h (Figure 10A,B).

**Figure 11:**
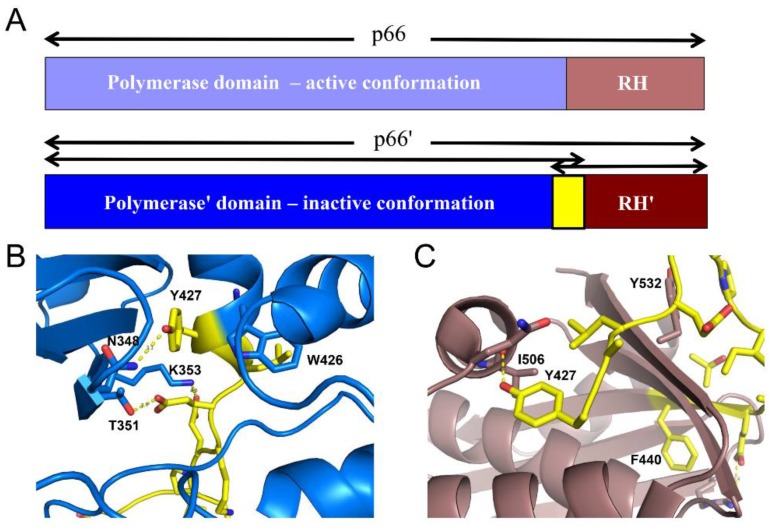
A tug-of-war for the residues at the N-terminus of the RNase H domain. (**A**) domain structure of the p66 and p66’ subunits, color coded as: polymerase (blue), RH (brown), and residues present in both polymerase’ and RH’ domains in p66’ (yellow); (**B**) Ribbon diagram of the p51 subunit of RT containing residues up to the cleavage site, (PDB ID: 1RTJ, [33]); (**C**) Ribbon diagram of the supernumerary RH domain. Color coding in panels B and C as in A. In p66*M*, the RH domain wins the tug-of-war, as the C-terminus of the connection unravels (Figure 3B). Formation of the dimer interface creates the cavity that binds αM’, facilitating the transfer of residues from RH’ to the polymerase’ (based on Figure 9 of [16]).

Maturation of the p66’ polymerase domain converts it from the *monomeric* form to the form observed in the p51 subunit of the heterodimer (Figure 3A). Formation of the RH:thumb’ interface completes the formation of the cavity that is filled by helix αM’ in the mature heterodimer. Initially, this helix is likely to be absent or distorted and to extend only to residue ~423 or 424. Over a longer time scale, the helix extends to include all of the C-terminal residues of the polymerase’, as well as additional residues that are contained in the RH’ domain, starting with Tyr427’. Extension of αM’ to include these residues thus competes with the RH’ domain for common residues, resulting in a molecular tug-of-war between the polymerase’ and RH’ domains of p66’ (Figure 11). In p66*M*, the outcome of this tug-of-war favors the folded RH domain, as illustrated in Figure 3B. The disordered orientation of the thumb domain removes part of the binding surface for helix αM, facilitating its unfolding so that it acts as a polymerase-RH domain linker. Completion of the binding cavity for helix αM’ occurs after dimerization and formation of the RH:thumb’ interface, altering the outcome of this competition for common residues (Figure 11). Thus, the inactive fold of the polymerase domain cannot coexist with the RH domain, while the active fold of the polymerase and the RH domain are both accommodated in the p66 subunit. Consistent with the critical role of helix αM’ in RT maturation, the corresponding amino acid sequence is very highly conserved in both native and drug-exposed patient populations [65].

### 4.3. Tyr427-Triggered RH Domain Unfolding

The competitive recruitment of common residues suggests a mechanism for subunit-selective RH domain unfolding. Zheng et al. [16] prepared an N-terminal deletion mutant of the RH domain: RH∆NT, lacking the two N-terminal residues Tyr427 and Gln428 and containing an L429M mutation at the new N-terminus. They showed that the mutated form remained capable of adopting a folded structure similar to that of the isolated wild-type domain; however, a urea denaturation study indicated that it was 2 kcal/mol less stable than the longer construct. Given a previous report of the stability of the RH domain of 3.7 kcal/mol [66], the loss of the two N-terminal residues represents a destabilization of RH’ by >50%.

**Figure 12:**
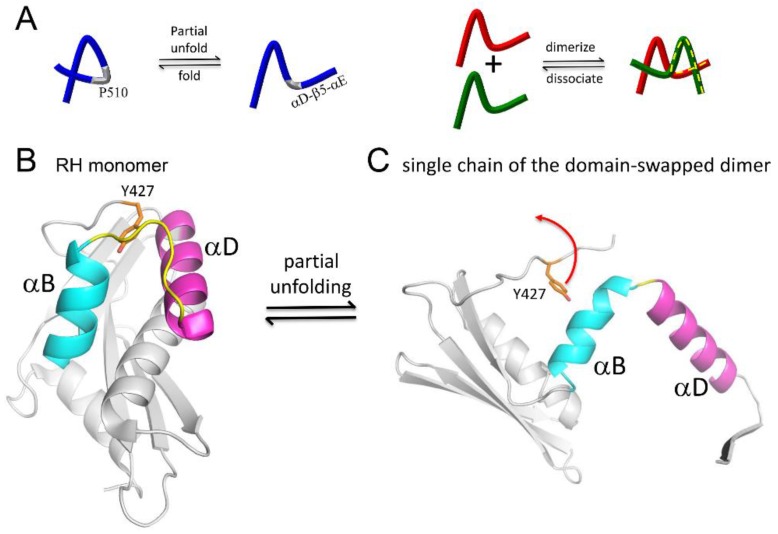
A domain-swapped structure of the RH domain reveals an unfolding pathway. (**A**) In the domain-swapped structure, the αB–αD loop becomes a hinge, allowing the released αD-β5-αE structural elements to interact with a second monomer; (**B**) the αB–αD loop (yellow) in the RH monomer adopts a strained conformation involving shortened helices that are able to extend by the incorporation of loop residues upon the formation of the domain-swapped form; (**C**) One of the monomer units of the domain-swapped dimer, showing the extended αB and αD helices and shortened hinge loop.

Further insight into the unfolding behavior of the RH domain has recently been obtained by investigation of an RH domain-swapped dimer [31]. Domain-swapped dimers allow observation of partially unfolded proteins that become stabilized as a result of the formation of a complementary set of interactions with a second molecule [67,68,69,70]. For the RT RH domain, such a metastable, partially unfolded state arises when the C-terminal αD-β5-αE structural elements separate from the N-terminus of the molecule (Figure 12). In making this transition, the B-D loop connecting helices B and D becomes a hinge that allows the C-terminus to be swapped between the two molecules of the dimer. As in other examples of this phenomenon, one important driver of this transition appears to be the strained conformation of this loop in the RH domain [68]. Separation of helices B and D allows the extension of each helix and the shortening of the B–D hinge loop from ~10 to ~2 residues. The stability of the extended helical structures increases the lifetime of the partially unfolded structure sufficiently to facilitate the formation of the domain-swapped dimer. The Tyr427 binding pocket is formed by the same two helices. Thus, instability of the Tyr427 binding pocket facilitates the excursion of this residue from its most stable conformation, which in turn facilitates helix separation.

Zheng et al. [31] suggest that structural instability of the RH domain facilitates “breathing” motions in which Tyr427 makes excursions from its binding pocket. In the isolated RH domain or the p66 monomer, these excursions are short lived; in p66/p66’, the polymerase' domain is able to exploit these excursions and capture Tyr427’ into helix αM’ (Figure 11C). Although a reversal of this process can occur, the RH’ domain must then persist in its destabilized state, lacking Tyr427’ and several following residues for a longer period of time, greatly increasing the likelihood of a more extensive unfolding of the RH’ domain. Thus, H/D amide exchange studies of the RH∆NT construct exhibit much higher exchange rates compared with the full RT domain, and the rate of unfolding required to interconvert monomer and domain-swapped dimer forms of RH∆NT is enhanced by more than two orders of magnitude [31].

Zheng et al. [31] observed time-dependent changes in the spectra of p66/p66’ in which several resonances of the thumb’ and connection’ domain that were barely visible in the initial homodimer spectra exhibited slow intensity increases on a time scale of hours. Concomitantly, resonances attributed to the RH’ domain of p66’ were observed to decay with similar time constants. These changes are incorporated in the model shown in Figure 10, in which residues are slowly transferred from RH’ to the connection' domain, with the destabilized RH’ domain unfolding, resulting in a loss of the observed RH’ resonances. The formation of helix αM’ in p66’ slowly fills the cavity formed by the connection and thumb’ domains, leading to time-dependent changes in resonances arising from residues in these domains.

### 4.4. Kinetic Barriers Stabilize Transient Intermediates

As outlined above, conversion of the monomer to the mature dimer involves passage through a series of relatively unstable structures that must persist for a sufficient length of time to engage in a new set of stabilizing interactions. Much as partially or incorrectly folded protein intermediates can become trapped in local energy minima, intermediates of the metamorphic transition can also become trapped by kinetic barriers. Conformational extension of the dissociated fingers/palm domains and recruitment of the disordered palm loop (Figure 5) interfere with rapid reassociation of the fingers/palm and connection domains, extending the lifetime of the domain-dissociated state, thus increasing the likelihood that more extensive structural rearrangements can occur. Formation of the small fingers:thumb interface provides short term stability of the thumb in a conformation that may facilitate formation of the palm:connection interface characteristic of the *extended* conformation (Figure 7). Subsequent to dimer formation, subunit-selective RH domain unfolding involves transient stabilization of the unfolded RH’, due to the extension of the αB and αD helices (Figure 12), facilitating the release of Tyr427’ so that it can be recruited by the polymerase domain [31]. As a result of the transfer of Tyr427’ to the polymerase, the destabilized RH’ domain is forced to persist for an extended time period, increasing the probability of RH’ unfolding. In the absence of this N-terminal residue transfer, the isolated RH domain and its domain-swapped form are stable [53,71,72].

## 5. Indirect Effects on Dimer Stability

### 5.1. The Structural Equilibrium of the Polymerase Domain

Stability of the RT heterodimer or its homodimer precursor has been shown to be sensitive to a variety of experimental conditions, ligands, and mutations. Since dimer formation follows domain rearrangement (as outlined above), many of these factors can exert their effects by influencing the earlier domain rearrangement process, rather than by directly influencing the monomer association step. Even perturbations that interfere with the subsequent subunit-specific RH domain unfolding step can influence the reversibility of the entire maturation pathway, and hence the fraction of p66 that is dimeric. Therefore, dimer stability provides a readout for multiple steps in the RT maturation pathway.

**Figure 13:**
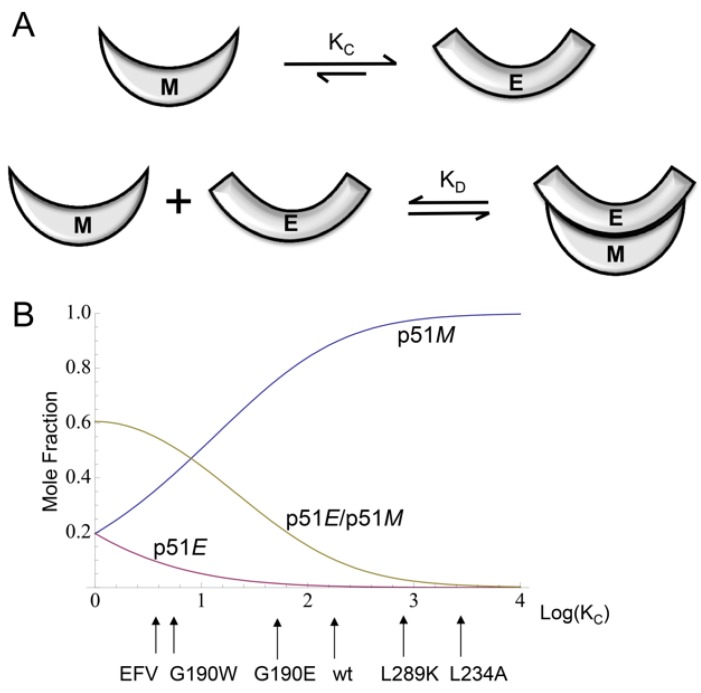
Conformational equilibria of the polymerase domain. (**A**) Schematic illustration of the two steps—domain rearrangement followed by monomer–monomer binding, required for homodimer formation. Formation of the initial dimer is proposed to involve a pair of molecules in the *E* and *M* conformations; (**B**) Calculated mole fractions of monomer and dimer species plotted as a function of Log[K_C_] for parameters K_D_ = 2.0 µM and total monomer concentration of 50 µM. Qualitative effects of the NNRTI efavirenz (EFV) and of several mutations that influence K_C_ and indirectly alter the apparent dimer stability are indicated on the horizontal axis. *E* and *M* represent the *extended* and *monomeric* conformations of the polymerase domain (based on Figure 9 of [25]).

Although the maturation of p66 represents the physiologically relevant process, the effects of experimental conditions, ligands, and many mutations on dimer stability can be more simply analyzed by considering the behavior of the isolated polymerase domain, p51. NMR studies of the Met-labeled and Ile-labeled polymerase domain indicate that the p51/p51’ homodimer exists as a structural heterodimer that does not exhibit the very slow conformational changes related to RH’ unfolding [24,25]. The domain isomerization/dimerization of p51 can be approximated by two equilibrium relations, one describing the equilibration of the monomer conformation with an *extended* conformation, and the second corresponding to dimerization of polymerase molecules in the *E* and *M* conformations (Figure 13):

(1) $$p51M\overset{K_{C}}{↔}p51E\;p51E+p51M\overset{K_{D}}{↔}p51E/p51M$$

These relations represent a minor variation from the previously described model, since it has now been determined that there are some structural differences between the p51*M* and p51*C* conformations (Figure 3A) [16]. The above equations predict that the dimerization of p51 can be described by an *apparent* dissociation constant K_d_^app^ that is dependent on both K_D_ and K_C_. For example, in the calculation shown in Figure 13, K_D_ is set at 2 µM in order to reveal the sensitivity of the monomer and dimer species to variations in K_C_. As outlined below, many of the perturbations that influence the monomer/dimer ratio act indirectly on dimer stability by influencing the K_C_ equilibrium. p66 dimerization and subsequent maturation follow a similar, but somewhat more complex set of reactions [24].

The effect of the L289K mutation on dimer stability provides the most compelling support for the model outlined above, since there appears to be no basis other than an effect on the *M/E* equilibrium for influencing dimer formation [25] (see below). Higher Mg^2+^ concentration and higher temperature have both been demonstrated to promote dimer formation and stability [25,55,73,74]. A solvent accessibility analysis of the active site aspartyl residues that bind the catalytic magnesium ion (Asp110, Asp185, and Asp186) indicates that they are all buried in the interior of the compact conformation, where they are involved in multiple hydrogen bond interactions; in the *extended* conformation, however, they become solvent exposed and form a cluster that facilitates Mg^2+^ ion chelation. Hence, the facilitating effect of Mg^2+^ on dimer formation is well described as resulting from conformationally-selective binding to the *extended* structure. Mg also exerts a direct effect on dimer formation similar to the effect of NaCl, as a result of its contribution to increased ionic strength. Similarly, the analysis shown in Figure 13 assumes that, as proposed by Wang et al. [48] and supported by the *monomeric* structure [16], the *compact* conformation is much more stable, so that the concentration ratio [*E*]/[*C*] << 1. Increasing the temperature will tend to increase the occupancy of the less-stable *extended* conformation, and hence is also predicted to exert an indirect effect favoring dimer formation, consistent with experimental observations [25,55,73,74].

### 5.2. Effects of NNRTIs on RT Maturation

**Figure 14:**
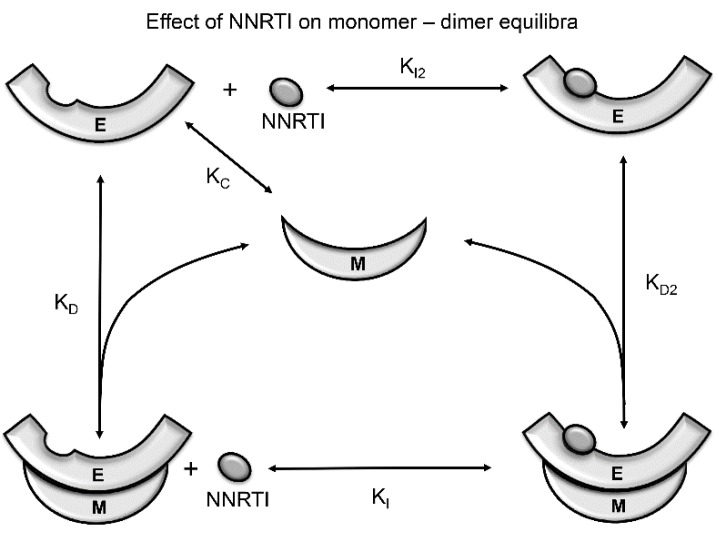
Effect of NNRTIs on RT conformational equilibria of the polymerase domain. The NNRTI binding site is present in the *extended* (*E*), but not the *compact* (*C*) or *monomeric* (*M*) conformation of the polymerase domain. It is assumed that the NNRTI can bind to the polymerase domain in the *E* conformation, whether it is present as a monomer (dissociation constant K_I2_) or a dimer (dissociation constant K_I_). The NNRTI pocket is dynamic, and so the NNRTI probably selects the form suitable for complex formation. Isomerization is believed to occur prior to dimer formation, so that a small amount of monomer in the *E* conformation is expected to be present. In either pathway, the binding energy of the ligand alters the conformational equilibrium, favoring the formation of the NNRTI–dimer complex (based on Figure 10 of [25]).

Non-nucleoside RT inhibitors have been identified as chemical enhancers of RT heterodimer formation [50,75,76], although in a few cases, the effect is absent or even destabilizing [77,78,79]. The NNRTI effect is thus paradoxical, often stabilizing the dimer structure, yet inhibiting activity as a consequence of local perturbations of active site residues [33,80]. Since the NNRTI binding site is present only in the *extended* polymerase conformation, NNRTIs will shift this equilibrium toward the *E* conformation as a result of conformationally-selective binding (Figure 14). Since the *extended* monomer is much less stable than the *compact* monomer (as demonstrated by the *monomer* structure), this additional enhancement of the *E/C* ratio will enhance dimer formation (Figure 13) [25]. Although the isolated p51*E* and p66*E* monomer species have not yet been observed, binding can in principle involve the *extended* conformations of either the monomer or dimer (Figure 14), and there is indirect evidence for the existence of active monomers [81,82].

The above analysis outlines a mechanism by which most NNRTIs can enhance apparent dimer stability, independent of the details of their specific interaction with the NNRTI binding site. Other mechanisms by which NNRTIs can influence the apparent dimer stability include: (1) perturbations of the structure of the dimer interface that are transmitted from the NNRTI binding pocket [50,83]; and (2) direct, competitive binding of NNRTIs at the subunit interface [78,79,84,85]. NNRTI-induced structural perturbations appear to involve alterations in the palm:fingers’ interface (Figure 9) [83,86]. Some hydrophobic NNRTIs may interact with residues at the subunit interface, similar to the effects of connection domain-derived peptides [85]. It is of course likely that some NNRTIs may influence dimer stability by several mechanisms. By stabilizing the dimer, NNRTIs are predicted to accelerate the RT maturation process. The reported ability of NNRTIs to stimulate intracellular polyprotein processing is consistent with premature activation of the HIV-1 protease by NNRTI-enhanced Gag–Pol multimerization through the embedded RT sequence [87].

In addition to the NNRTIs, other active site ligands, including dsDNA, hybrid RNA–DNA, and metal–nucleotide complexes are each expected to stabilize the dimer as a result of selective binding to the *extended* structure of the polymerase domain [47,88,89]. These interactions will in general not be synergistic, since the ligands often select different active site conformations, resulting in negative binding cooperativity (e.g., [80,90]). In addition, double-stranded nucleotides can contact both subunits of the RT heterodimer, providing an additional basis for stabilization.

### 5.3. Mutational Effects on RT Maturation

As in the case of symmetric multimers such as HIV protease, the metamorphic RT polymerase domain must accommodate a pair of mutations for each mutational variation in the p66 precursor. This constraint can be mitigated in protein regions with differential sensitivity to mutations. For example, the β7’–β8’ loop on p66’ (or p51) plays an important role at the dimer interface (Figure 9), while the same segment on p66 is a solvent-exposed loop that is more tolerant to mutational variation [61]. Alternatively, the palm loop segment on p66’ is disordered and presumably highly tolerant of mutational variations, while in the p66 subunit this segment is structurally important and forms part of the primer grip motif. Thus, the asymmetric structure of the dimer can result in mutational effects that are strongly subunit-dependent [61,91,92].

Numerous studies have shown that RT mutations can influence dimer stability [41,54,60,62,91,92,93,94,95,96,97]. As in the example of the NNRTIs discussed above, these effects can involve both direct effects resulting from perturbations of the dimer interface, or indirect effects that involve other steps of the maturation process. The large number of drug-resistance mutations that have been identified correspond to variations that fall within the tolerance limits of the maturation pathway. The greater stability of the polymerase in the inactive conformation observed in the p51 subunit suggests that this subunit may be able to function as a mutational buffer. Thus, a mutation that is functionally advantageous in the p66 subunit but destabilizes p51 may still be tolerated, due to the ability of p51 to absorb the structural disruption introduced by the mutation.

Among the most interesting mutational perturbations are those that influence dimer stability but are located far from the dimer interface. The reduction in dimer stability produced by the L289K mutation provides perhaps the clearest example of a mutation for which the dimer stabilizing effect cannot result from a perturbation of the dimer interface, and hence must function via an indirect influence on dimer stability [25,60]. For the RT heterodimer, dimer destabilization was found to result when the mutation was on the p66 subunit rather than on the p51 subunit [60]. This is a particularly surprising result, since Leu289 on p51 is located at the dimer interface, while Leu289 on p66 is located far from the interface. This effect is thus opposite to that predicted on the basis of a direct destabilization of the dimer interface. The effect of this mutation can be explained based on a perturbation of the *E/M* conformer ratio [25]. In p66*M*, Leu289 is solvent exposed, while in the structure of apo RT which serves as a model for p66*E*, Leu289 is located in a small fingers:thumb interface and makes hydrophobic contacts with fingers residues Phe61, Ile63, and Leu74 in the predominant closed conformation (Figure 7). Hence, the non-conservative L289K mutation is predicted to decrease the ratio of *E/M* species, indirectly reducing the dimer concentration (Figure 13B). In addition to providing the only reasonable explanation for the basis of the L289K effect on dimer stability, this analysis also provides insight into why apo RT might adopt a very unusual closed fingers–thumb conformation that can only bind its RNA–DNA or DNA–DNA substrate if the fingers–thumb domains separate. Thus, the closed conformation provides an additional stabilizing effect on the relatively unstable *E* conformation that helps to orient the domains in order to facilitate maturation (Figure 7B).

Alanine scanning studies of the primer grip region have identified several mutations, including W229A, G231A, and L234A that reduce heterodimer association [92,93,94]. As outlined above, this region of the palm domain—which is not initially present in the monomer—plays a central role in the *M→E* conversion by forming a β-sheet that forms part of the palm:connection interface (Figure 6). The short β-sheet is stabilized by hydrophobic interactions between Leu234 and Trp229, Trp239, and Tyr232. The L234A and W229A mutations will not only reduce these interactions substantially, but the intrinsic α-helical preference of Ala should further destabilize the β-sheet structure. Consistent with this analysis, the non-conservative L234D mutation is reported to almost completely block heterodimer formation, while the more conservative L234F mutation that preserves the hydrophobic character of the Leu reduces but does not block dimer formation [54]. Gly231 supports the formation of the β-turn connecting the first two strands (β12 and β13 following the nomenclature of Wang et al. [48]) of this sheet [98]. Thus, the W239A, G231A, and L274A mutations would be expected to interfere with the conformational transitions required to form the new palm:connection interface that is present in the *E* conformation (Figure 6). Consistent with the above analysis, the β-sheet residues noted above are highly conserved, even in patient populations treated with RT inhibitors [99]. These mutations will produce the same kind of indirect domain rearrangement-dependent interference with dimer formation as that produced by the palm loop deletion mutation. In contrast with the palm loop deletion, it is often possible to form at least some dimer by using sufficiently favorable conditions (e.g., high concentration and high salt concentration). Reported drug resistance mutations at these positions are generally more conservative (e.g., F227L, F227C, L234I, L234F, L234I, and P236L substitutions) [100,101,102,103]. More extreme variations that might confer greater resistance to NNRTI binding may be selected against as a result of an unacceptable level of interference with the maturation pathway.

Tachedjian et al. [92] further identified additional p66 mutations that could partially or fully overcome the dimerization defect produced by the Leu234 mutation. Since the assay used evaluates p66 dimer formation with p51, it is expected to depend on the ability of p66*M* to adopt the *extended* conformation, necessary for formation of the p66*E*/p51*C* dimer. Four identified rescue mutations include two active site aspartyl residues, D110G and D186V, and two connection domain tryptophan residues, W402R and W406R. For each of these residues, there is a large difference in solvent exposure between the two subunits. Analysis of side chain exposure for structure 1DLO with the program VADAR [104] for residues 110, 186, 402, and 406 gives solvent accessibility fractions of 0.40, 0.16, 0.57, and 0.51, respectively, in the *extended* p66 conformation; and values of 0.07, 0.05, 0.06, and 0.00, respectively, for the *compact* p51 conformation. Similar values are obtained for the monomer (PDB ID: 4KSE). Structural analysis indicates that the buried Asp110 and Asp186 residues in p51 make multiple H-bond and salt bridge contacts with other residues, while the buried Trp402 and Trp406 stabilize the *compact* conformation due to hydrophobic interactions. Trp406 Nε-H is also involved in stabilizing H-bond interactions in the compact p51 subunit. The role of the aspartyl residues of the YMDD motif in stabilizing the *compact* conformation of p51 has also been demonstrated by studies of Mulky et al. [105]. Alternatively, we estimate the effects of the mutations in the *extended* conformation as neutral or slightly destabilizing with the additional possibility that W402R and W406R may be capable of dimer stabilization due to the formation of inter-subunit H-bond interactions. Consequently, while the L234A mutation is expected to lower the *E/C* conformational ratio of p66, the rescue mutations are expected to increase this ratio, primarily due to destabilization of the *compact* conformation. In summary, the effects of the dimerization inhibiting and rescue mutations studied by Tachedjian et al. [92] appear to be well explained by the conformational selection model described by Zheng et al. [25] and illustrated in Figure 13.

**Figure 15:**
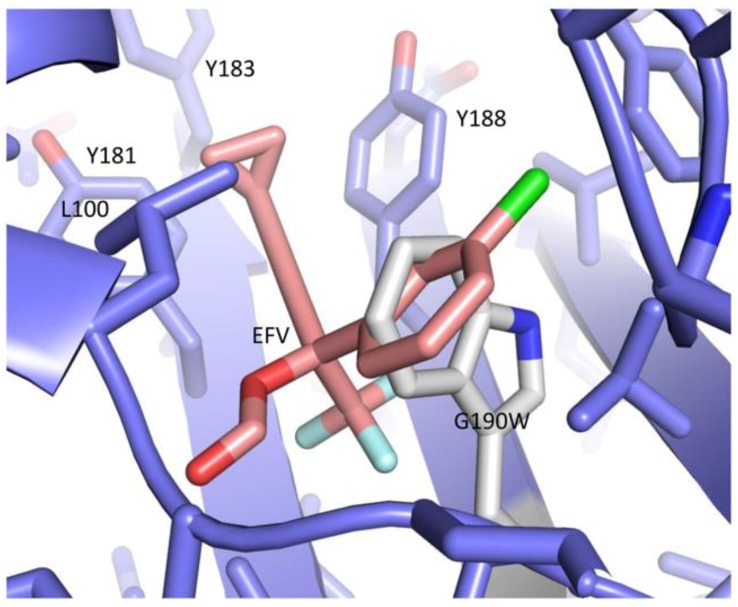
Accommodation of the G190W mutation by the NNRTI site. Ribbon diagram of the RT–EFV complex (PDB ID: 1FK9, [32]) containing a G190W mutation modeled using Pymol (DeLano Scientific, Palo Alto, CA, USA). The NNRTI site in the p66*E* structure is in principle able to accommodate the large Trp190 side chain in the same region that accommodates EFV. Introduction of the same mutation into the compact p51 structure results is substantial steric conflicts (not shown). The p66 palm domain is shown in blue, EFV is beige, and the mutated G190W residue is gray. Since the NNRTI site is present only in the *extended* (*E*) conformation, the G190W mutation increases the *E/C* ratio, promoting dimer formation as illustrated in Figure 14.

Non-nucleoside RT inhibitor resistance due to mutation of Gly190—located in the NNRTI binding pocket—involves a significant expansion of side chain volume in a densely-packed region of the protein. The G190A, G190S, G190E, and G190Q substitutions have all been reported to confer NNRTI drug resistance [106,107,108,109,110,111]. Figueiredo et al. [91] carried out extensive subunit-specific mutational studies of Gly190, and found that most p66 subunit substitutions significantly enhanced dimer formation, while mutations in p51 were destabilizing. Although Gly190 is located far from the subunit interface in p66*M* and p66*C*, introduction of the larger side chains in the *compact* conformer will lead to local structural disruption that may be transmitted further into the structure, perturbing the dimer interface. The corresponding region in p66 in the apo structure is somewhat less crowded, but more importantly, exhibits substantial intrinsic flexibility that allows the accommodation of large NNRTIs. Movement of side chains in the NNRTI site of the *E* conformation can thus allow the accommodation of mutations requiring substantially greater side chain volume. It was determined that the most extreme side chain expansion (corresponding to a G190W mutation) was able to enhance dimer stability nearly as effectively as the NNRTI efavirenz (EFV) [91]. Consistent with this observation, modeling the G190W substitution into the structure of the RT–EFV complex, PDB ID: 1FK9, [32] shows that the tryptophan side chain can occupy much of the same region that is filled by EFV (Figure 15). Consequently, the G190W mutation can be considered as a mimic of an NNRTI, and is expected to be able to enhance dimer formation by increasing the *E/C* conformer ratio, similar to the mechanism discussed above for NNRTIs (Figure 14). This example further suggests that mutations of other residues in or near the NNRTI binding site that significantly increase side chain volume may have a similar effect of promoting dimer formation by increasing the ratio of the *E/C* conformers.

### 5.4. Mutational Perturbation of the Polymerase-RH Tug-of-War

As outlined above, subunit-specific RH domain unfolding is the final step in the maturation of the p66/p66’ homodimer. Mutations that significantly destabilize the RH domain can result in premature unfolding and proteolysis that effectively uncouples this step from dimer formation. Precursors containing mutations at the Phe440–Tyr441 cleavage site or in the segment between the domain N-terminal Tyr427 residue and the cleavage site have been reported to result in excessive formation of p51 and low or even absent levels of the RT heterodimer [39,53,112]. However, p66 monomers that are not proteolyzed may still be able to undergo a maturation process if formation of the intra- and inter-subunit interfaces of the dimer are sufficient to overcome the destabilizing effects of the mutation.

Abram et al. identified T477A as a rescue mutation for F440V and several other mutations that destabilize the RH domain and lead to premature processing [38,113]. Based on the tug-of-war model (Figure 11), we suggest that the rescue mutation provides sufficient additional RH domain stability to restore the conformational equilibrium of p66*M* illustrated in Figure 3B, in which the RH domain remains folded so that unfolding occurs only subsequent to dimer formation. Although the basis for this effect is not completely clear, Thr477 is located on the first α-helix of the RH domain, and the stabilizing effect of the T477A mutation may result from replacing the Thr residue, which has a weak propensity for β-strand formation, with Ala, which has a strong propensity to form α-helical structure [98,114]. Thus, consistent with the RH domain unfolding model outlined above, the Thr477 residue has evolved not for maximum stability—which would be enhanced by an Ala residue at this position—but to provide just enough stability so that the loss of a few N-terminal residues is sufficient to permit domain unfolding.

## 6. Maturation within the Virion

The RT maturation processes outlined above are mostly accomplished within the virion after it has separated from the host cell. Various lines of evidence indicate that the viral polyproteins exist primarily as a linear assembly of folded proteins and protein domains [87,115], adopting a beads-on-a-string type of structure analogous to that of the p66 monomer (Figure 2B and Figure 4). This type of arrangement also shields potential cleavage sites in the folded domains from HIV protease and form proteases present in the host cell. Dimerization of the polyprotein is facilitated by anchoring of the myristoylated polyprotein in the membrane [116,117], and becomes more probable at the higher concentrations present in the virion—initial concentrations of Gag and Gag-pro-Pol are estimated as 3.6 mM and 0.2 mM, respectively [118], facilitating the formation of the active HIV protease dimer necessary for viral maturation.

**Figure 16:**
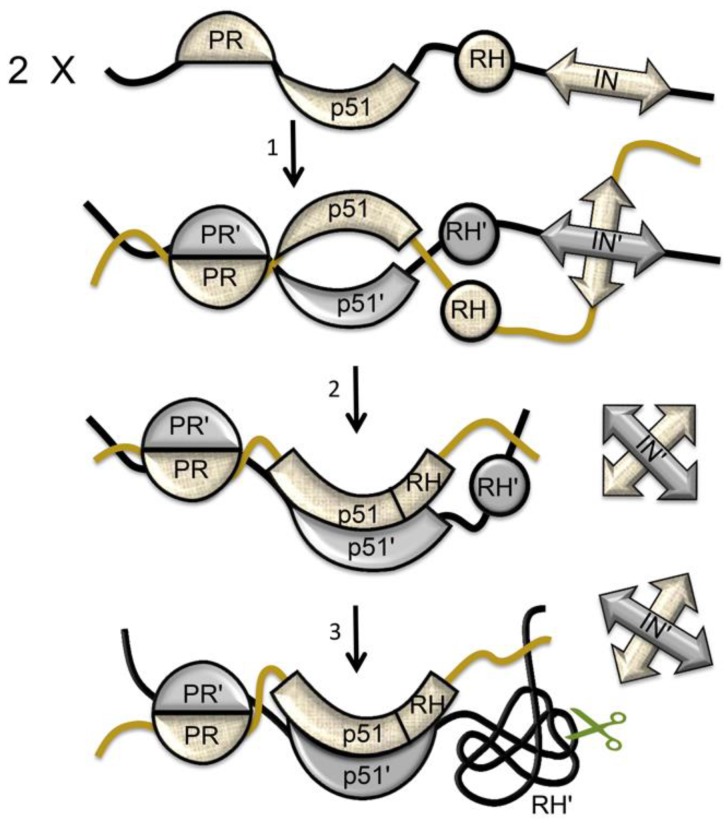
Polyprotein-dependent cooperative dimerization. Solvent-exposed dimer interfaces in the folded regions of Gag-pro-Pol presumably provide the initial nucleation sites for dimer formation (**1**). Formation of the initial polyprotein homodimer facilitates p51 dimerization, since a p51 binding partner is positioned nearby to capture the (rare) isomerized p51 structure (**2**). Early cleavage releases the integrase (IN) dimer, followed by the transfer of residues from RH’ to p51’, leading to the destabilization and unfolding of RH’, and to the exposure of the RH’ cleavage site, indicated by the green scissors (**3**). Cleavage of protease (PR)-p51 occurs near the end of proteolytic processing.

The structures of the RT heterodimer and its p66/p66’ homodimer precursor (Figure 10A) are arranged so that the two p66 chains are positioned in a roughly parallel orientation, consistent with the existence of a multiprotein dimer (Figure 16). Multiple studies indicate that in the absence of ligands, the C-terminal helix E of RH is largely disordered, freeing as many as 22 residues on each subunit to act as linkers to an IN dimer [66,71,119,120,121]. Alternatively, although there are five disordered residues at the N-terminus of the p51 monomer (PDB ID: 4KSE, [16]), formation of a PR-RT linker would require additional unraveling of the N-termini. This is readily accomplished; the N-termini of both RT subunits contain proline-rich sequences that run along the protein surface and can easily be recruited to form linker segments that connect to the pair of C-termini of the HIV-1 protease dimer without producing significant destabilization (unpublished results).

The polyprotein structure of pro-Pol results in cooperative dimerization, in which formation of an initial homodimer facilitates more extensive dimer formation (Figure 16). Since in order to dimerize, the polymerase (p51) must undergo a prior structural isomerization, dimerization of p66 is likely to occur subsequent to dimerization of the PR and IN components of the polyprotein (Figure 16). According to studies of polyprotein processing by Pettit et al. [122], the RH-IN bond is cleaved at a relatively early stage (stage 2 on the 1–5 scale used in this reference), the p51-RH bond on an intermediate time scale (stage 3), and the PR-RT bond is cleaved near the end of proteolytic processing. It is thus likely that much of the polyprotein processing involves PR dimers that remain linked to RT dimers, although the greater activity of the cleaved protease dimer may lead to a disproportionate contribution [123]. These results are consistent with the studies summarized in this review, indicating that processing of the p51-RH bond is dependent on RH unfolding, which in turn depends on dimer formation. Experimental studies supporting the cooperativity of dimer formation have been reported by Figueiredo et al. [87] and Sudo et al. [117], who showed that the addition of EFV or other dimer-inducing NNRTIs to HIV-transfected cells led to premature PR activation, resulting in intracellular processing of the polyprotein and a decrease in viral particle production. In this case, the order of dimer formation differs from that illustrated in Figure 16. Thus, the EFV induces isomerization of the polymerase domain and dimer formation, probably by the mechanism shown in Figure 14. Reverse transcriptase dimerization then facilitates dimerization of PR and IN, as well as supporting subunit-selective RH’ unfolding—required prior to p51-RH cleavage. NNRTI-induced cooperative polyprotein dimerization leading to protease activation and premature maturation may also contribute to the greater potency of NNRTIs (compared with nucleoside reverse transcriptase inhibitors (NRTIs)) in reducing cell-to-cell HIV transmission [124].

In addition to the higher polyprotein concentration in the virion, other interactions are likely to facilitate RT dimerization. There is evidence that a pre-initiation reverse transcription complex (RTC) that includes viral RNA (vRNA), tRNA(Lys3), and RT is formed within the virion [125,126]. In general, formation of an early RTC with immature forms of the p66/p66’ homodimer is likely to accelerate RT maturation by stabilizing dimeric forms of the enzyme and reducing dissociation. For example, Cabodevilla et al. [49] have reported that the addition of poly(rA)/T_20_ lowers the equilibrium dissociation constant for p66 by about 30-fold. Formation of a complex with double-stranded RNA (dsRNA) formed from vRNA annealed with tRNA(Lys3) requires an extended conformation of the polymerase domain, which will stabilize the dimer and presumably accelerate the maturation of the p66’ subunit shown in Figure 10. Annealing of the vRNA with tRNA(Lys3) is catalyzed by the nucleocapsid (NC) protein [127,128], a process that may additionally be facilitated by the interaction of RT with NC [129]. In addition to stabilization of the extended p66 polymerase conformation in the p66 subunit, the dsRNA probably also interacts with several residues on the p66’ subunit—Lys390, Lys395, and Glu396—that form part of the RNase H primer grip motif [130,131]. These interactions can work cooperatively with polyprotein dimerization in facilitating RT maturation.

In addition to supporting dimer formation, the high concentration of p66 within the virion can compensate for many p66 mutations that reduce but do not prevent dimer formation. Thus, Wapling et al. [40] observed that the W401A mutation that reduces dimer stability interfered with the processing of p66 to p51 in cell lysates, while RT processing in the virion experienced a much weaker perturbation. This difference was suggested to result from the ability of the much higher virion concentration of p66 to overcome the destabilizing effects of the W401A mutation. In our experience with in vitro systems, the dimer-blocking effects of most single residue mutations can be overcome at sufficiently high concentrations, or by favorable experimental conditions ([24,25] and unpublished results).

The virion pH is not well regulated, making the intra-virion pH subject to variations as a result of changes in the viral medium (e.g., [132]). Since viral maturation relies on the activity of an aspartyl protease (which exhibits a pH optimum in the range of pH 4–6 [133]), and since RNA degradation is base catalyzed [134], it would seem advantageous for the virion to maintain an acidic pH. It is likely that the maturation steps outlined above have evolved to work optimally at the virion pH. Low pH values have been reported to significantly destabilize RT as a result of subunit dissociation [73]. Formation of the vRNA–tRNA(Lys3)–RT complex may provide sufficient stability to overcome the effects of a variable virion pH. Among the interactions expected to be most sensitive to small pH variations near neutrality, the polymerase:RH interface may be sensitive to the protonation state of His361 (Figure 8B).

## 7. Maturation of HIV-2 Reverse Transcriptase

**Figure 17:**
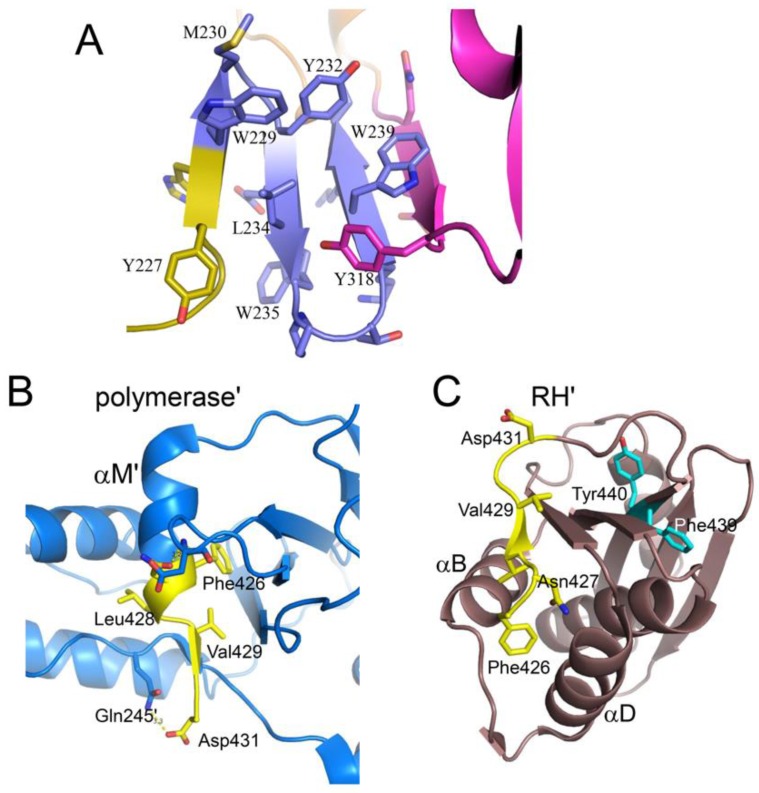
Structural features of HIV-2 RT. (**A**) Ribbon diagram showing the p66 subunit β-sheet in HIV-2 RT formed from palm and connection residues as part of the maturation process; color code: palm loop (gold); palm (blue); connection (magenta); (**B**) Ribbon diagram of the HIV-2 RT p51 subunit (blue) showing the residues Phe426–Phe439 that are also present in the RH domain in yellow; (**C**) Ribbon diagram of the HIV-2 RH domain (brown), with the residues from Phe426–Phe439 shown in yellow. These structural features suggest a maturation process for HIV-2 RT that is similar to the process described for HIV-1 RT.

Reverse transcriptases derived from HIV-1 and HIV-2 show only ~60% sequence identity [135]; however, there are sufficient structural similarities to support the conclusion that the maturation of HIV-2 RT follows the same general pathway as that of the HIV-1 enzyme [34]. Similar to the HIV-1 enzyme, the p51 subunit of HIV-2 RH (and presumably the HIV-2 p66 monomer) includes a disordered palm loop (residues 212–228) that becomes the first strand of the β-sheet that forms part of the palm:connection interface in the p66 subunit (Figure 17A). Residue Leu234 in the β-sheet at the palm:connection interface similarly plays a central role in creating a hydrophobic network that stabilizes the β-sheet. The N-terminal residue of the HIV-2 RH domain is Phe rather than Tyr, and there are six resolved residues present in both the inactively-folded polymerase domain and the RH domain, consistent with a similar tug-of-war mechanism for subunit-selective unfolding of RH’ (Figure 17B,C). The p51 subunit of HIV-2 RT includes a short extended structure following helix αM’.

## 8. Reverse Transcriptase Maturation as an Attractive Drug Target

The coding economy that results from reliance on a metamorphic RT comes at the cost of constraints on mutational variability and reliance on a complex maturation pathway. Recent NMR, crystallographic, and modeling studies have provided initial insights into this pathway, and suggest that maturation may be an attractive target for therapeutic intervention. In contrast, nucleoside drugs that target mature RT can also interfere with activities of host polymerases, and in some cases have been shown to inhibit the mitochondrial polymerase, leading to associated HIV lipodystrophy [20]. In the absence of an effective HIV vaccine and cure, it remains imperative that the antiretroviral drug pipeline contains new classes of HIV inhibitors that are active against circulating drug-resistant strains [136].

The feasibility of targeting the final, subunit-specific RH domain unfolding step using an RH-active site-directed ligand has recently been demonstrated [31]. As discussed above, maturation inhibitors that interfere with domain rearrangement and the formation of new interface contacts indirectly interfere with dimer formation, which has been a popular research target [85,137,138,139]. Alternatively, the development of compounds that interfere with dimer formation by competitively binding to residues at the dimer interface is extremely challenging, due to its large size. This difficulty is exacerbated by the high concentrations of p66 precursors in the virion. There is also evidence for dimer formation involving a larger segment of the polyprotein [87], further limiting the feasibility of directly targeting the dimer interface. However, several steps in the maturation process may be more easily targeted with small molecule ligands.

The RH:thumb’ interface—which is proposed to be absent in the initial homodimer structure (Figure 10A)—represents an attractive target for interfering with RT maturation. Agopian et al. [140] have developed several thumb-derived peptides that inhibit RT maturation and block viral maturation at subnanomolar concentrations. However, this peptide apparently does not bind in such a way that it significantly interferes with the stability of the mature RT heterodimer or alters the open/closed ratio of the fingers:thumb [90]. Wendeler et al. [141] and Chung et al. [142] have described vinylogous ureas that interfere with RNase H activity, possibly by interacting with the p51 thumb' domain and altering the dimer interface [141,143], and Masaoka et al. [144] have described thumb domain-targeted thienopyrimidinones that are predicted to bind near the RH:thumb’ interface and destabilize the heterodimer, decreasing the melting temperature by 5 °C. At the present time, no structural data is available for the corresponding complexes with RT or the isolated thumb domain, and it is unclear to what extent these inhibitors may interfere with the formation of the RH:thumb’ interface.

## Figures and Tables

**Table 1:** Nomenclature.

Abbreviations	Definitions
Polymerase domain	Residues
Fingers (F)	1–84; 119–154
Palm (P)^1^	85–118; 155–241
Thumb (T)	242–313
Connection (C)	314–426
RH—RNase H domain	427–560
	Description/example
p51*E*^2^	*Extended* (*E*) structure of the active polymerase domain; corresponds to residues 1–426 of the p66 subunit of the reverse transcriptase (RT) heterodimer (Figure 1A)
p51*C*	*Compact* (*C*) structure of the polymerase domain; observed in the p51 subunit of RT (Figure 1A and Figure 2B)
p51*M*	*Monomeric* (*M*) p51 structure, observed in crystallized p51∆PL (Figure 3A)
p66*E*^2^	p66 structure in the RT heterodimer (Figure 2A)
p66*C*^3^	p66 structure containing an inactively-folded polymerase domain linked to a ribonuclease H (RH) domain
p66*M*	*M* structure of p66 (Figure 3B)
p66/p66’	Homodimer in which the p66 subunit conformation approximates p66*E*, and p66’ matures from an initial p66*M*’-like conformation to a final conformation containing a p51*C’* subunit linked to an unfolded RH’ domain

^1^ F/P refers to the non-dissociable fingers/palm domains corresponding to residues 1–241; ^2^ Conformational variability of the *E* structures includes the alternate open or closed fingers–thumb conformation and the flexible non-nucleoside RT inhibitor (NNRTI) binding pocket; ^3^ The p66*C* structure containing the inactive polymerase domain undergoes larger conformational transitions during maturation, developing from an initial p66*M*-like conformation to form the p51 subunit of the mature heterodimer linked to an unfolded RH domain [24].

**Table 2:** Crystal structures referenced.

PDB ID	Description	Reference
1DLO	Unliganded human immunodeficiency virus 1 (HIV-1) RT heterodimer	[27]
1S9E	RT-NNRTI complex in which the often disordered residues of the p51 palm loop are observed	[28]
4KSE	p51∆PL monomer, lacking the disordered palm loop—unable to adopt an *E* structure	[16]
1HAR	RT216, a construct that includes the fingers and most of the palm domain	[29]
1HRH	Isolated RH domain	[23]
3K2P	Isolated RH-active site inhibitor complex	[30]
5DZM	Domain-swapped RH dimer—each RH chain corresponds to a partially unfolded monomer	[31]
1FK9	RT heterodimer–efavirenz (EFV) complex	[32]
1RTJ	RT heterodimer in which the p51 subunit extends to the Phe440 cleavage site residue	[33]
1MU2	Unliganded HIV-2 RT heterodimer	[34]

## References

[1] Lindahl T. (1993). Instability and decay of the primary structure of DNA. Nature.

[2] Holmes E.C. (2003). Error thresholds and the constraints to RNA virus evolution. Trends Microbiol..

[3] Smith R.A., Loeb L.A., Preston B.D. (2005). Lethal mutagenesis of HIV. Virus Res..

[4] Roberts J.D., Bebenek K., Kunkel T.A. (1988). The Accuracy of Reverse-Transcriptase from Hiv-1. Science.

[5] Sierra S., Kupfer B., Kaiser R. (2005). Basics of the virology of HIV-1 and its replication. J. Clin. Virol..

[6] Belshaw R., Pybus O.G., Rambaut A. (2007). The evolution of genome compression and genomic novelty in RNA viruses. Genome Res..

[7] Biswas P., Jiang X., Pacchia A.L., Dougherty J.P., Peltz S.W. (2004). The human immunodeficiency virus type 1 ribosomal frameshifting site is an invariant sequence determinant and an important target for antiviral therapy. J. Virol..

[8] Jacks T., Power M.D., Masiarz F.R., Luciw P.A., Barr P.J., Varmus H.E. (1988). Characterization of Ribosomal Frameshifting in Hiv-1 Gag-Pol Expression. Nature.

[9] Mouzakis K.D., Lang A.L., Vander Meulen K.A., Easterday P.D., Butcher S.E. (2013). HIV-1 frameshift efficiency is primarily determined by the stability of base pairs positioned at the mRNA entrance channel of the ribosome. Nucleic Acids Res..

[10] Geyer M., Fackler O.T., Peterlin B.M. (2001). Structure-function relationships in HIV-1 Nef. EMBO Rep..

[11] Malim M.H., Emerman M. (2008). HIV-1 accessory proteins—Ensuring viral survival in a hostile environment. Cell Host Microbe.

[12] Schubert U., Bour S., Ferrer-Montiel A.V., Montal M., Maldarell F., Strebel K. (1996). The two biological activities of human immunodeficiency virus type 1 Vpu protein involve two separable structural domains. J. Virol..

[13] Katz R.A., Skalka A.M. (1994). The Retroviral Enzymes. Annu. Rev. Biochem..

[14] Wlodawer A., Miller M., Jaskólski M., Sathyanarayana B.K., Baldwin E., Weber I.T., Selk L.M., Clawson L., Schneider J., Kent S.B. (1989). Conserved Folding in Retroviral Proteases—Crystal-Structure of a Synthetic Hiv-1 Protease. Science.

[15] Hare S., Gupta S.S., Valkov E., Engelman A., Cherepanov P. (2010). Retroviral intasome assembly and inhibition of DNA strand transfer. Nature.

[16] Zheng X., Pedersen L.C., Gabel S.A., Mueller G.A., Cuneo M.J., DeRose E.F., Krahn J.M., London R.E. (2014). Selective unfolding of one Ribonuclease H domain of HIV reverse transcriptase is linked to homodimer formation. Nucleic Acids Res..

[17] Jochmans D. (2008). Novel HIV-1 reverse transcriptase inhibitors. Virus Res..

[18] Sarafianos S.G., Marchand B., Das K., Himmel D.M., Parniak M.A., Hughes S.H., Arnold E. (2009). Structure and Function of HIV-1 Reverse Transcriptase: Molecular Mechanisms of Polymerization and Inhibition. J. Mol. Biol..

[19] Menendez-Arias L. (2008). Mechanisms of resistance to nucleoside analogue inhibitors of HIV-1 reverse transcriptase. Virus Res..

[20] Lewis W., Day B.J., Copeland W.C. (2003). Mitochondrial toxicity of NRTI antiviral drugs: An integrated cellular perspective. Nat. Rev. Drug Discov..

[21] Ding J., Jacobo-Molina A., Tantillo C., Lu X., Nanni R.G., Arnold E. (1994). Buried surface analysis of HIV-1 reverse transcriptase p66/p51 heterodimer and its interaction with dsDNA template/primer. J. Mol. Recognit..

[22] Kohlstaedt L.A., Wang J., Friedman J.M., Rice P.A., Steitz T.A. (1992). Crystal-Structure at 3.5 Angstrom Resolution of Hiv-1 Reverse-Transcriptase Complexed with an Inhibitor. Science.

[23] Davies J.F., Hostomska Z., Hostomsky Z., Jordan S.R., Matthews D.A. (1991). Crystal-Structure of the Ribonuclease-H Domain of Hiv-1 Reverse-Transcriptase. Science.

[24] Zheng X., Perera L., Mueller G.A., DeRose E.F., London R.E. (2015). Asymmetric conformational maturation of HIV-1 reverse transcriptase. eLife.

[25] Zheng X.H., Mueller G.A., Cuneo M.J., DeRose E.F., London R.E. (2010). Homodimerization of the p51 Subunit of HIV-1 Reverse Transcriptase. Biochemistry.

[26] Kensch O., Restle T., WoÈhrl B.M., Goody R.S., Steinhoff H.J. (2000). Temperature-dependent equilibrium between the open and closed conformation of the p66 subunit of HIV-1 reverse transcriptase revealed by site-directed spin labelling. J. Mol. Biol..

[27] Hsiou Y., Ding J., Das K., Clark A.D., Hughes S.H., Arnold E. (1996). Structure of unliganded HIV-1 reverse transcriptase at 2.7 angstrom resolution: Implications of conformational changes for polymerization and inhibition mechanisms. Structure.

[28] Das K., Clark A.D., Lewi P.J., Heeres J., de Jonge M.R., Koymans L.M.H., Vinkers H.M., Daeyaert F., Ludovici D.W., Kukla M.J. (2004). Roles of conformational and positional adaptability in structure-based design of TMC125-R165335 (etravirine) and related non-nucleoside reverse transcriptase inhibitors that are highly potent and effective against wild-type and drug-resistant HIV-1 variants. J. Med. Chem..

[29] Unge T., Knight S., Bhikhabhai R., Lovgren S., Dauter Z., Wilson K., Strandberg B. (1994). 2.2 A resolution structure of the amino-terminal half of HIV-1 reverse transcriptase (fingers and palm subdomains). Structure.

[30] Himmel D.M., Maegley K.A., Pauly T.A., Bauman J.D., Das K., Dharia C., Clark A.D., Ryan K., Hickey M.J., Love R.A. (2009). Structure of HIV-1 Reverse Transcriptase with the Inhibitor beta-Thujaplicinol Bound at the RNase H Active Site. Structure.

[31] Zheng X., Pedersen L.C., Gabel S.A., Mueller G.A., DeRose E.F., London R.E. (2016). Unfolding the HIV-1 reverse transcriptase RNase H domain - how to lose a molecular tug-of-war. Nucleic Acids Res..

[32] Ren J., Milton J., Weaver K.L., Short S.A., Stuart D.I., Stammers D.K. (2000). Structural basis for the resilience of efavirenz (DMP-266) to drug resistance mutations in HIV-1 reverse transcriptase. Structure.

[33] Esnouf R., Ren J.S., Ross C., Jones Y., Stammers D., Stuart D. (1995). Mechanism of Inhibition of Hiv-1 Reverse-Transcriptase by Nonnucleoside Inhibitors. Nat. Struct. Biol..

[34] Ren J., Bird L.E., Chamberlain P.P., Stewart-Jones G.B., Stuart D.I., Stammers D.K. (2002). Structure of HIV-2 reverse transcriptase at 2.35-angstrom resolution and the mechanism of resistance to non-nucleoside inhibitors. Proc. Natl. Acad. Sci. USA.

[35] Schulze T., Nawrath M., Moelling K. (1991). Cleavage of the Hiv-1 P66 Reverse-Transcriptase Rnase-H by the P9 Protease in vitro Generates Active P15 Rnase-H. Arch. Virol..

[36] Tomasselli A.G., Sarcich J.L., Barrett L.J., Reardon I.M., Howe W.J., Evans D.B., Sharma S.K., Heinrikson R.L. (1993). Human-Immunodeficiency-Virus Type-1 Reverse-Transcriptase and Ribonuclease-H as Substrates of the Viral Protease. Protein Sci..

[37] Chattopadhyay D., Evans D.B., Deibel M.R., Vosters A.F., Eckenrode F.M., Einspahr H.M., Hui J.O., Tomasselli A.G., Zurcher-Neely H.A., Heinrikson R.L. (1992). Purification and Characterization of Heterodimeric Human-Immunodeficiency-Virus Type-1 (Hiv-1) Reverse-Transcriptase Produced by in vitro Processing of P66 with Recombinant Hiv-1 Protease. J. Biol. Chem..

[38] Slack R.L., Spiriti J., Ahn J., Parniak M.A., Zuckerman D.M., Ishima R. (2015). Structural integrity of the ribonuclease H domain in HIV-1 reverse transcriptase. Proteins-Struct. Funct. Bioinform..

[39] Navarro J.M., Damier L., Boretto J., Priet S., Canard B., Quérat G., Sire J. (2001). Glutamic residue 438 within the protease-sensitive subdomain of HIV-1 reverse transcriptase is critical for heterodimer processing in viral particles. Virology.

[40] Wapling J., Moore K.L., Sonza S., Mak J., Tachedjian G. (2005). Mutations that abrogate human immunodeficiency virus type 1 reverse transcriptase dimerization affect maturation of the reverse transcriptase heterodimer. J. Virol..

[41] Sluis-Cremer N., Arion D., Abram M.E., Parniak M.A. (2004). Proteolytic processing of an HIV-1 pol polyprotein precursor: Insights into the mechanism of reverse transcriptase p66/p51 heterodimer formation. Int. J. Biochem. Cell Biol..

[42] Murzin A.G. (2008). Biochemistry—Metamorphic proteins. Science.

[43] Bryan P.N., Orban J. (2010). Proteins that switch folds. Curr. Opin. Struct. Biol..

[44] Doublie S., Sawaya M.R., Ellenberger T. (1999). An open and closed case for all polymerases. Struct Fold. Des..

[45] Sawaya M.R., Pelletier H., Kumar A., Wilson S.H., Kraut J. (1994). Crystal-Structure of Rat DNA-Polymerase-Beta—Evidence for a Common Polymerase Mechanism. Science.

[46] Wong J.H., Fiala K.A., Suo Z., Ling H. (2008). Snapshots of a Y-family DNA polymerase in replication: Substrate-induced conformational transitions and implications for fidelity of Dpo4. J. Mol. Biol..

[47] Lansdon E.B., Samuel D., Lagpacan L., Brendza K.M., White K.L., Hung M., Ray A.S. (2010). Visualizing the Molecular Interactions of a Nucleotide Analog, GS-9148, with HIV-1 Reverse Transcriptase-DNA Complex. J. Mol. Biol..

[48] Wang J., Smerdon S.J., Jäger J., Kohlstaedt L.A., Rice P.A., Friedman J.M., Steitz T.A. (1994). Structural Basis of Asymmetry in the Human-Immunodeficiency-Virus Type-1 Reverse-Transcriptase Heterodimer. Proc. Natl. Acad. Sci. USA.

[49] Cabodevilla J.F., Odriozola L., Santiago E., Martínez-Irujo J.J. (2001). Factors affecting the dimerization of the p66 form of HIV-1 reverse transcriptase. Eur. J. Biochem..

[50] Venezia C.F., Howard K.J., Ignatov M.E., Holladay L.A., Barkley M.D. (2006). Effects of efavirenz binding on the subunit equilibria of HIV-1 reverse transcriptase. Biochemistry.

[51] Jacobo-Molina A., Arnold E. (1991). Hiv Reverse-Transcriptase Structure-Function-Relationships. Biochemistry.

[52] Ding J.P., Das K., Hsiou Y., Sarafianos S.G., Clark A.D., Jacobo-Molina A., Arnold E. (1998). Structure and functional implications of the polymerase active site region in a complex of HIV-1 RT with a double-stranded DNA template-primer and an antibody Fab fragment at 2.8 angstrom resolution. J. Mol. Biol..

[53] Hostomska Z., Matthews D.A., Davies J.F., Nodes B.R., Hostomsky Z. (1991). Proteolytic Release and Crystallization of the Rnase-H Domain of Human-Immunodeficiency-Virus Type-1 Reverse-Transcriptase. J. Biol. Chem..

[54] Pattyn E., Lavens D., Van der Heyden J., Verhee A., Lievens S., Lemmens I., Tavernier J. (2008). MAPPIT (MAmmalian Protein-Protein Interaction Trap) as a tool to study HIV reverse transcriptase dimerization in intact human cells. J. Virol. Meth..

[55] Divita G., Restle T., Goody R.S. (1993). Characterization of the Dimerization Process of Hiv-1 Reverse-Transcriptase Heterodimer Using Intrinsic Protein Fluorescence. FEBS Lett..

[56] Venezia C.F., Meany B.J., Braz V.A., Barkley M.D. (2009). Kinetics of Association and Dissociation of HIV-1 Reverse Transcriptase Subunits. Biochemistry.

[57] Paris K.A., Haq O., Felts A.K., Das K., Arnold E., Levy R.M. (2009). Conformational Landscape of the Human Immunodeficiency Virus Type 1 Reverse Transcriptase Non-Nucleoside Inhibitor Binding Pocket: Lessons for Inhibitor Design from a Cluster Analysis of Many Crystal Structures. J. Med. Chem..

[58] Tantillo C., Ding J., Jacobo-Molina A., Nanni R.G., Boyer P.L., Hughes S.H., Arnold E. (1994). Locations of Anti-Aids Drug-Binding Sites and Resistance Mutations in the 3-Dimensional Structure of Hiv-1 Reverse-Transcriptase—Implications for Mechanisms of Drug-Inhibition and Resistance. J. Mol. Biol..

[59] Chou P.Y., Fasman G.D. (1979). Prediction of Beta-Turns. Biophys. J..

[60] Goel R., Beard W.A., Kumar A., Casas-Finet J.R., Strub M.P., Stahl S.J., Becerra S.P. (1993). Structure-Function Studies of Hiv-1(1) Reverse-Transcriptase—Dimerization-Defective Mutant L289k. Biochemistry.

[61] Pandey P.K., Kaushik N., Singh K., Sharma B., Upadhyay A.K., Kumar S., Pandey V.N. (2002). Insertion of a small peptide of six amino acids into the beta 7-beta 8 loop of the p51 subunit of HIV-1 reverse transcriptase perturbs the heterodimer and affects its activities. BMC Biochemistry.

[62] Mulky A., Vu B.C., Conway J.A., Hughes S.H., Kappes J.C. (2007). Analysis of amino acids in the beta 7-beta 8 loop of human immunodeficiency virus type 1 reverse transcriptase for their role in virus replication. J. Mol. Biol..

[63] Sharma S.K., Fan N.S., Evans D.B. (1994). Human-Immunodeficiency-Virus Type-1 (Hiv-1) Recombinant Reverse-Transcriptase—Asymmetry in P66 Subunits of the P66/P66 Homodimer. FEBS Lett..

[64] Sharaf N.G., Poliner E., Slack R.L., Christen M.T., Byeon I.J. L., Parniak M.A., Ishima R. (2014). The p66 immature precursor of HIV-1 reverse transcriptase. Proteins.

[65] Santos A.F., Lengruber R.B., Soares E.A., Jere A., Sprinz E., Martinez A.M., Soares M.A. (2008). Conservation patterns of HIV-1 RT connection and RNase H domains: Identification of new mutations in NRTI-treated patients. PLoS ONE.

[66] Kern G., Handel T., Marqusee S. (1998). Characterization of a folding intermediate from HIV-1 ribonuclease H. Protein Sci..

[67] Byeon I.J., Louis J.M., Gronenborn A.M. (2004). A captured folding intermediate involved in dimerization and domain-swapping of GB1. J. Mol. Biol..

[68] Newcomer M.E. (2002). Protein folding and three-dimensional domain swapping: A strained relationship?. Curr. Opin. Struct. Biol..

[69] Rousseau F., Schymkowitz J., Itzhaki L.S. (2012). Implications of 3D domain swapping for protein folding, misfolding and function. Adv. Exp. Med. Biol..

[70] Rousseau F., Schymkowitz J.W., Itzhaki L.S. (2003). The unfolding story of three-dimensional domain swapping. Structure.

[71] Pari K., Mueller G.A., DeRose E.F., Kirby T.W., London R.E. (2003). Solution structure of the RNase H domain of the HIV-1 reverse transcriptase in the presence of magnesium. Biochemistry.

[72] Christen M.T., Menon L., Myshakina N.S., Ahn J., Parniak M.A., Ishima R. (2012). Structural basis of the allosteric inhibitor interaction on the HIV-1 reverse transcriptase RNase H domain. Chem. Biol. Drug. Des..

[73] Divita G., Rittinger K., Geourjon C., Deléage G., Goody R.S. (1995). Dimerization Kinetics of Hiv-1 and Hiv-2 Reverse-Transcriptase—A 2-Step Process. J. Mol. Biol..

[74] Rowley G.L., Ma Q.F., Bathurst I.C., Barr P.J., Kenyon G.L. (1990). Stabilization and Activation of Recombinant Human Immunodeficiency Virus-1 Reverse Transcriptase-P66. Biochem. Biophys. Res. Commun..

[75] Tachedjian G., Orlova M., Sarafianos S.G., Arnold E., Goff S.P. (2001). Nonnucleoside reverse transcriptase inhibitors are chemical enhancers of dimerization of the HIV type 1 reverse transcriptase. Proc. Natl. Acad. Sci. USA.

[76] Tachedjian G., Moore K.L., Goff S.P., Sluis-Cremer N. (2005). Efavirenz enhances the proteolytic processing of an HIV-1 pol polyprotein precursor and reverse transcriptase homodimer formation. FEBS Lett..

[77] Sluis-Cremer N., Arion D., Parniak M.A. (2002). Destabilization of the HIV-1 reverse transcriptase dimer upon interaction with N-acyl hydrazone inhibitors. Mol. Pharmacol..

[78] Camarasa M.J., Velazquez S., San-Felix A., Perez-Perez M.J., Bonache M.C., Castro S.D. (2006). TSAO derivatives, inhibitors of HIV-1 reverse transcriptase dimerization: Recent progress. Curr. Pharm. Des..

[79] Sluis-Cremer N., Hamamouch N., San Félix A., Velazquez S., Balzarini J., Camarasa M.J. (2006). Structure-activity relationships of [2′,5′-bis-O-(tert-butyldimethylsilyl)-beta-D-ribofuranosyl]-3′-spiro-5″-(4″-amino-1″,2″-oxathiole-2″,2″-dioxide) thymine derivatives as inhibitors of HIV-1 reverse transcriptase dimerization. J. Med. Chem..

[80] Das K., Martinez S.E., Bauman J.D., Arnold E. (2012). HIV-1 reverse transcriptase complex with DNA and nevirapine reveals non-nucleoside inhibition mechanism. Nat. Struct. Mol. Biol..

[81] Pandey P.K., Kaushik N., Talele T.T., Yadav P.N.S., Pandey V.N. (2001). Insertion of a peptide from MuLV RT into the connection subdomain of HIV-1 RT results in a functionally active chimeric enzyme in monomeric conformation. Mol. Cell. Biochem..

[82] Tachedjian G., Radzio J., Sluis-Cremer N. (2005). Relationship between enzyme activity and dimeric structure of recombinant HIV-1 reverse transcriptase. Proteins-Struct. Funct. Bioinform..

[83] Das K., Bauman J.D., Rim A.S., Dharia C., Clark A.D., Camarasa M.J., Arnold E. (2011). Crystal Structure of tert-Butyldimethylsilyl-spiroaminooxathioledioxide-thymine (TSAO-T) in Complex with HIV-1 Reverse Transcriptase (RT) Redefines the Elastic Limits of the Non-nucleoside Inhibitor-Binding Pocket. J. Med. Chem..

[84] Sluis-Cremer N., Dmitrienko G.I., Balzarini J., Camarasa M.J., Parniak M.A. (2000). Human immunodeficiency virus type 1 reverse transcriptase dimer destabilization by 1-{spiro[4″-amino-2″,2″-dioxo-1″,2″-oxathiole-5″,3′-[2′,5′-bis-O-(tert-butyldimethylsilyl)-beta-D-ribofuranosyl]]}-3-ethylthymine. Biochemistry.

[85] Divita G., Restle T., Goody R.S., Chermann J.C., Baillon J.G. (1994). Inhibition of Human-Immunodeficiency-Virus Type-1 Reverse-Transcriptase Dimerization Using Synthetic Peptides Derived from the Connection Domain. J. Biol. Chem..

[86] Nissley D.V., Radzio J., Ambrose Z., Sheen C.W., Hamamouch N., Moore K.L., Sluis-Cremer N. (2007). Characterization of novel non-nucleoside reverse transcriptase (RT) inhibitor resistance mutations at residues 132 and 135 in the 51 kDa subunit of HIV-1 RT. Biochem. J..

[87] Figueiredo A., Moore K.L., Mak J., Sluis-Cremer N., de Bethune M.P., Tachedjian G. (2006). Potent nonnucleoside reverse transcriptase inhibitors target HIV-1 Gag-Pol. PLoS Pathog..

[88] Das K., Sarafianos S.G., Clark A.D., Boyer P.L., Hughes S.H., Arnold E. (2007). Crystal structures of clinically relevant Lys103Asn/Tyr181Cys double mutant HIV-1 reverse transcriptase in complexes with ATP and non-nucleoside inhibitor HBY 097. J. Mol. Biol..

[89] Huang H., Chopra R., Verdine G.L., Harrison S.C. (1998). Structure of a covalently trapped catalytic complex of HIV-1 reverse transcriptase: implications for drug resistance. Science.

[90] Zheng X.H., Mueller G.A., DeRose E.F., London R.E. (2013). Protein-Mediated Antagonism between HIV Reverse Transcriptase Ligands Nevirapine and MgATP. Biophys. J..

[91] Figueiredo A., Zelina S., Sluis-Cremer N., Tachedjian G. (2008). Impact of residues in the nonnucleoside reverse transcriptase inhibitor binding pocket on HIV-1 reverse transcriptase heterodimer stability. Curr. HIV Res..

[92] Tachedjian G., Aronson H.E.G., Goff S.P. (2000). Analysis of mutations and suppressors affecting interactions between the subunits of the HIV type 1 reverse transcriptase. Proc. Natl. Acad. Sci. USA.

[93] Ghosh M., Jacques P.S., Rodgers D.W., Ottman M., Darlix J.L., Le Grice S.F. (1996). Alterations to the primer grip of p66 HIV-1 reverse transcriptase and their consequences for template-primer utilization. Biochemistry.

[94] Wohrl B.M., Krebs R., Thrall S.H., Le Grice S.F., Scheidig A.J., Goody R.S. (1997). Kinetic analysis of four HIV-1 reverse transcriptase enzymes mutated in the primer grip region of p66—Implications for DNA synthesis and dimerization. J. Biol. Chem..

[95] Mulky A., Sarafianos S.G., Jia Y., Arnold E., Kappes J.C. (2005). Identification of amino acid residues in the human immunodeficiency virus type-1 reverse transcriptase tryptophan-repeat motif that are required for subunit interaction using infectious virions. J. Mol. Biol..

[96] Tachedjian G., Aronson H.E.G., de los Santos M., Seehra J., McCoy J.M., Goff S.P. (2003). Role of residues in the tryptophan repeat motif for HIV-1 reverse transcriptase dimerization. J. Mol. Biol..

[97] Auwerx J., Van Nieuwenhove J., Rodríguez-Barrios F., de Castro S., Velázquez S., Ceccherini-Silberstein F., Balzarini J. (2005). The N137 and P140 amino acids in the p51 and the P95 amino acid in the p66 subunit of human immunodeficiency virus type I (HIV-1) reverse transcriptase are instrumental to maintain catalytic activity and to design new classes of anti-HIV-1 drugs. FEBS Lett..

[98] Malkov S.N., Živković M.V., Beljanski M.V., Hall M.B., Zarić S.D. (2008). A reexamination of the propensities of amino acids towards a particular secondary structure: Classification of amino acids based on their chemical structure. J. Mol. Model..

[99] Ceccherini-Silberstein F., Gago F., Santoro M., Gori C., Svicher V., Rodríguez-Barrios F., Balzarini J. (2005). High sequence conservation of human immunodeficiency virus-type 1 reverse transcriptase under drug pressure despite the continuous appearance of mutations. J. Virol..

[100] Fujiwara T., Sato A., El-Farrash M., Miki S., Abe K., Isaka Y., Sugimoto H. (1998). S-1153 inhibits replication of known drug-resistant strains of human immunodeficiency virus type 1. Antimicrob. Agents Chemother..

[101] Feng M.Z., Wang D., Grobler J.A., Hazuda D.J., Miller M.D., Lai M.T. (2015). In Vitro Resistance Selection with Doravirine (MK-1439), a Novel Nonnucleoside Reverse Transcriptase Inhibitor with Distinct Mutation Development Pathways. Antimicrob. Agents Chemother..

[102] Sato A., Hammond J., Alexander T.N., Graham J.P., Binford S., Sugita K.I., Patick A.K. (2006). In vitro selection of mutations in human immunodeficiency virus type 1 reverse transcriptase that confer resistance to capravirine, a novel nonnucleoside reverse transcriptase inhibitor. Antivir. Res..

[103] Lu M.Q., Felock P.J., Munshi V., Hrin R.C., Wang Y.J., Yan Y., Williams T.M. (2012). Antiviral Activity and In Vitro Mutation Development Pathways of MK-6186, a Novel Nonnucleoside Reverse Transcriptase Inhibitor. Antimicrob. Agents Chemother..

[104] Willard L., Ranjan A., Zhang H., Monzavi H., Boyko R.F., Sykes B.D., Wishart D.S. (2003). VADAR: A web server for quantitative evaluation of protein structure quality. Nucleic Acids Res..

[105] Mulky A., Sarafianos S.G., Arnold E., Wu X., Kappes J.C. (2004). Subunit-specific analysis of the human immunodeficiency virus type 1 reverse transcriptase in vivo. J. Virol..

[106] Bacheler L., Jeffrey S., Hanna G., D’Aquila R., Wallace L., Logue K., Baker D. (2001). Genotypic correlates of phenotypic resistance to efavirenz in virus isolates from patients failing nonnucleoside reverse transcriptase inhibitor therapy. J. Virol..

[107] Kleim J.P., Bender R., Kirsch R., Meichsner C., Paessens A., Rösner M., Schneweis K.E. (1995). Preclinical Evaluation of Hby-097, a New Nonnucleoside Reverse-Transcriptase Inhibitor of Human-Immunodeficiency-Virus Type-1 Replication. Antimicrob. Agents Chemother..

[108] Kolomeets A.N., Varghese V., Lemey P., Bobkova M.R., Shafer R.W. (2014). A uniquely prevalent nonnucleoside reverse transcriptase inhibitor resistance mutation in Russian subtype A HIV-1 viruses. AIDS.

[109] Neogi U., Anita S.H.E.T., Shamsundar R., Ekstrand M.L. (2011). Selection of nonnucleoside reverse transcriptase inhibitor-associated mutations in HIV-1 subtype C: Evidence of etravirine cross-resistance. AIDS.

[110] Paolucci S., Baldanti F., Campanini G., Cancio R., Belfiore A., Maga G., Gerna G. (2007). NNRTI-selected mutations at codon 190 of human immunodeficiency virus type 1 reverse transcriptase decrease susceptibility to stavudine and zidovudine. Antivir. Res..

[111] Wang J., Dykes C., Domaoal R.A., Koval C.E., Bambara R.A., Demeter L.M. (2006). The HIV-1 reverse transcriptase mutants G190S and G190A, which confer resistance to non-nucleoside reverse transcriptase inhibitors, demonstrate reductions in RNase H activity and DNA synthesis from tRNA(Lys,3) that correlate with reductions in replication efficiency. Virology.

[112] Abram M.E., Parniak M.A. (2005). Virion instability of human immunodeficiency virus type 1 reverse transcriptase (RT) mutated in the protease cleavage site between RT p51 and the RT RNase H domain. J. Virol..

[113] Abram M.E., Sarafianos S.G., Parniak M.A. (2010). The mutation T477A in HIV-1 reverse transcriptase (RT) restores normal proteolytic processing of RT in virus with Gag-Pol mutated in the p51-RNH cleavage site. Retrovirology.

[114] Chou P.Y., Fasman G.D. (1978). Empirical Predictions of Protein Conformation. Annu. Rev. Biochem..

[115] Deshmukh L., Ghirlando R., Clore G.M. (2015). Conformation and dynamics of the Gag polyprotein of the human immunodeficiency virus 1 studied by NMR spectroscopy. Proc. Natl. Acad. Sci. USA.

[116] Li H., Dou J., Ding L., Spearman P. (2007). Myristoylation is required for human immunodeficiency virus type 1 gag-gag multimerization in mammalian cells. J. Virol..

[117] Sudo S., Haraguchi H., Hirai Y., Gatanaga H., Sakuragi J.I., Momose F., Morikawa Y. (2013). Efavirenz Enhances HIV-1 Gag Processing at the Plasma Membrane through Gag-Pol Dimerization. J. Virol..

[118] Konnyu B., Sadiq S.K., Turányi T., Hírmondó R., Müller B., Kräusslich H.G., Müller V. (2013). Gag-Pol Processing during HIV-1 Virion Maturation: A Systems Biology Approach. PLoS Comput. Biol..

[119] Zheng X., Mueller G.A., DeRose E.F., London R.E. (2012). Metal and ligand binding to the HIV-RNase H active site are remotely monitored by Ile556. Nucleic Acids Res..

[120] Powers R., Clore G.M., Stahl S.J., Wingfield P.T., Gronenborn A. (1992). Analysis of the Backbone Dynamics of the Ribonuclease-H Domain of the Human-Immunodeficiency-Virus Reverse-Transcriptase Using N-15-Relaxation Measurements. Biochemistry.

[121] Mueller G.A., Pari K., DeRose E.F., Kirby T.W., London R.E. (2004). Backbone dynamics of the RNase H domain of HIV-1 reverse transcriptase. Biochemistry.

[122] Pettit S.C., Lindquist J.N., Kaplan A.H., Swanstrom R. (2005). Processing sites in the human immunodeficiency virus type 1 (HIV-1) Gag-Pro-Pol precursor are cleaved by the viral protease at different rates. Retrovirology.

[123] Louis J.M., Clore G.M., Gronenborn A.M. (1999). Autoprocessing of HIV-1 protease is tightly coupled to protein folding. Nat. Struct. Biol..

[124] Agosto L.M., Zhong P., Munro J., Mothes W. (2014). Highly Active Antiretroviral Therapies Are Effective against HIV-1 Cell-to-Cell Transmission. PLoS Pathog..

[125] Warrilow D., Stenzel D., Harrich D. (2007). Isolated HIV-1 core is active for reverse transcription. Retrovirology.

[126] Kleiman L., Jones C.P., Musier-Forsyth K. (2010). Formation of the tRNALys packaging complex in HIV-1. FEBS Lett..

[127] Lapadat-Tapolsky M., Pernelle C., Borie C., Darlix J.L. (1995). Analysis of the nucleic acid annealing activities of nucleocapsid protein from HIV-1. Nucleic Acids Res..

[128] Thomas J.A., Gorelick R.J. (2008). Nucleocapsid protein function in early infection processes. Virus Res..

[129] Druillennec S., Caneparo A., de Rocquigny H., Roques B.P. (1999). Evidence of interactions between the nucleocapsid protein NCp7 and the reverse transcriptase of HIV-1. J. Biol. Chem..

[130] Delviks-Frankenberry K.A., Nikolenko G.N., Barr R., Pathak V.K. (2007). Mutations in human immunodeficiency virus type 1 RNase H primer grip enhance 3-Azido-3’-deoxythymidine resistance. J. Virol..

[131] Sarafianos S.G., Das K., Tantillo C., Clark A.D., Ding J., Whitcomb J.M., Arnold E. (2001). Crystal structure of HIV-1 reverse transcriptase in complex with a polypurine tract RNA : DNA. EMBO J..

[132] Padilla-Parra S., Marin M., Gahlaut N., Suter R., Kondo N., Melikyan G.B. (2013). Fusion of Mature HIV-1 Particles Leads to Complete Release of a Gag-GFP-Based Content Marker and Raises the Intraviral pH. PLoS ONE.

[133] Ido E., Han H.P., Kezdy F.J., Tang J. (1991). Kinetic-Studies of Human-Immunodeficiency-Virus Type-1 Protease and Its Active-Site Hydrogen-Bond Mutant A28s. J. Biol. Chem..

[134] Li Y.F., Breaker R.R. (1999). Kinetics of RNA degradation by specific base catalysis of transesterification involving the 2’-hydroxyl group. J. Am. Chem. Soc..

[135] Hizi A., Tal R., Shaharabany M., Loya S. (1991). Catalytic Properties of the Reverse Transcriptases of Human Immunodeficiency Viruses Type-1 and Type-2. J. Biol. Chem..

[136] Waheed A.A., Tachedjian G. (2016). Why Do We Need New Drug Classes for HIV Treatment and Prevention?. Curr. Top. Med. Chem..

[137] Sluis-Cremer N., Tachedjian G. (2002). Modulation of the oligomeric structures of HIV-1 retroviral enzymes by synthetic peptides and small molecules. Eur. J. Biochem..

[138] Andreola M.L. (2009). Therapeutic Potential of Peptide Motifs Against HIV-1 Reverse Transcriptase and Integrase. Curr. Pharm. Des..

[139] Restle T., Muller B., Goody R.S. (1990). Dimerization of Human-Immunodeficiency-Virus Type-1 Reverse-Transcriptase—A Target for Chemotherapeutic Intervention. J. Biol. Chem..

[140] Agopian A., Gros E., Aldrian-Herrada G., Bosquet N., Clayette P., Divita G. (2009). A New Generation of Peptide-based Inhibitors Targeting HIV-1 Reverse Transcriptase Conformational Flexibility. J. Biol. Chem..

[141] Wendeler M., Lee H.F., Bermingham A., Miller J.T., Chertov O., Bona M.K., Gotte M. (2008). Vinylogous Ureas as a Novel Class of Inhibitors of Reverse Transcriptase-Associated Ribonuclease H Activity. ACS Chem. Biol..

[142] Chung S.M., Wendeler M., Rausch J.W., Beilhartz G., Gotte M., O’Keefe B.R., Le Grice S.F. (2010). Structure-Activity Analysis of Vinylogous Urea Inhibitors of Human Immunodeficiency Virus-Encoded Ribonuclease H. Antimicrob. Agents Chemother..

[143] Chung S.M., Miller J.T., Johnson B.C., Hughes S.H., Le Grice S.F.J. (2012). Mutagenesis of Human Immunodeficiency Virus Reverse Transcriptase p51 Subunit Defines Residues Contributing to Vinylogous Urea Inhibition of Ribonuclease H Activity. J. Biol. Chem..

[144] Masaoka T., Chung S., Caboni P., Rausch J.W., Wilson J.A., Taskent-Sezgin H., Le Grice S.F. (2013). Exploiting Drug-Resistant Enzymes as Tools To Identify Thienopyrimidinone Inhibitors of Human Immunodeficiency Virus Reverse Transcriptase-Associated Ribonuclease H. J. Med. Chem..

